# A histone deacetylase 7‐derived peptide promotes vascular regeneration via facilitating 14‐3‐3γ phosphorylation

**DOI:** 10.1002/stem.3122

**Published:** 2020-01-29

**Authors:** Junyao Yang, Ana Moraga, Jing Xu, Yue Zhao, Peiyi Luo, Ka Hou Lao, Andriana Margariti, Qiang Zhao, Wei Ding, Gang Wang, Min Zhang, Lei Zheng, Zhongyi Zhang, Yanhua Hu, Wen Wang, Lisong Shen, Alberto Smith, Ajay M Shah, Qian Wang, Lingfang Zeng

**Affiliations:** ^1^ School of Cardiovascular Medicine and Sciences, King's College – London British Heart Foundation Centre of Excellence, Faculty of Life Science and Medicine, King's College London London UK; ^2^ Department of Clinical Laboratory Xinhua Hospital, Shanghai Jiao Tong University School of Medicine Shanghai People's Republic of China; ^3^ Institute of Bioengineering, Queen Mary University of London London UK; ^4^ Centre for Experimental Medicine Queen's University Belfast Belfast UK; ^5^ State Key Laboratory of Medicinal Chemical Biology, Key Laboratory of Bioactive Materials, Ministry of Education College of Life Sciences, Nankai University Tianjin People's Republic of China; ^6^ Department of Emergency Medicine The Second Affiliated Hospital, Xi'an Jiaotong University Xi'an People's Republic of China; ^7^ Southern Medical University Guangzhou People's Republic of China

**Keywords:** histone deacetylase 7, peptide, phosphorylation, short open reading frame, vascular progenitor cell

## Abstract

Histone deacetylase 7 (HDAC7) plays a pivotal role in the maintenance of the endothelium integrity. In this study, we demonstrated that the intron‐containing *Hdac7* mRNA existed in the cytosol and that ribosomes bound to a short open reading frame (sORF) within the 5′‐terminal noncoding area of this *Hdac7* mRNA in response to vascular endothelial growth factor (VEGF) stimulation in the isolated stem cell antigen‐1 positive (Sca1^+^) vascular progenitor cells (VPCs). A 7‐amino acid (7A) peptide has been demonstrated to be translated from the sORF in Sca1^+^‐VPCs in vitro and in vivo. The 7A peptide was shown to receive phosphate group from the activated mitogen‐activated protein kinase MEKK1 and transfer it to 14‐3‐3 gamma protein, forming an MEKK1‐7A‐14‐3‐3γ signal pathway downstream VEGF. The exogenous synthetic 7A peptide could increase Sca1^+^‐VPCs cell migration, re‐endothelialization in the femoral artery injury, and angiogenesis in hind limb ischemia. A *Hd7‐7sFLAG* transgenic mice line was generated as the loss‐of‐function model, in which the 7A peptide was replaced by a FLAG‐tagged scrabbled peptide. Loss of the endogenous 7A impaired Sca1^+^‐VPCs cell migration, re‐endothelialization of the injured femoral artery, and angiogenesis in ischemic tissues, which could be partially rescued by the addition of the exogenous 7A/7Ap peptide. This study provides evidence that sORFs can be alternatively translated and the derived peptides may play an important role in physiological processes including vascular remodeling.

## Graphical abstract

VEGF induced the translation of a 7‐amino acid peptide (7A) from Hdac7 mRNA in mouse adventitial Sca1+ vascular progenitor cells, which formed a novel MEKK1‐7A‐14‐3‐3γ signal pathway downstream VEGF and contributed to vascular injury repair and neo‐angiogenesis in ischemic tissues. The deficiency of 7A peptide (7A‐null mice) retarded these processes, which could be rescued by exogenous 7A peptide.

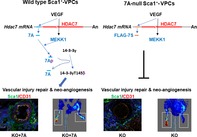

**Significance statement**

Histone deacetylase 7 (HDAC7) plays a pivotal role in the maintenance of the endothelial integrity. Short open reading frames (sORFs) exist within the 5′‐terminal noncoding area of Hdac7 mRNA. It remains unclear whether these sORFs contribute to HADC7 functions. In the present study, we demonstrated that a 7‐amino acid (7A) peptide could be translated from a sORF. This peptide could act as phosphate group carrier, forming a novel signal transduction pathway, the MEKK1‐7A‐14‐3‐3γ pathway, downstream vascular endothelial growth factor. The novel signal pathway may be involved in vessel wall resident stem/progenitor cell activation and vascular remodeling.

## 1 INTRODUCTION

Endothelial injury is a key event in the development of cardiovascular diseases, whereas the regeneration of damaged endothelium is essential for the protection against such diseases.^1^, ^2^ Growth factors and the activation of local stem/progenitor cells capable to differentiate toward endothelial cells play a pivotal role in this process.^3^, ^4^ Vascular progenitor cells (VPCs), which are mainly located in the adventitia, also contribute to the repair of the injured endothelium via migration toward the endothelium and differentiation into the endothelial cell (EC) lineage.^5^, ^6^

In eukaryotic species, a single gene can produce multiple messenger RNA (mRNA) molecules through the alternative splicing process, giving rise to different protein variants with different, even opposite, functions.^7^ Recent studies have shown that the translation of a peptide/protein can be initiated from different start codon AUG or CUG (up to 15%) codons within a single mRNA molecule.^8^ Thus, a single mRNA molecule may encode different peptides or proteins, which makes the genetic information stored in the gene sequence even vaster.

Histone deacetylases (HDACs) are a family of enzymes that remove acetyl groups from N‐acetylated lysine residues on histones and are involved in gene transcriptional regulation through modulating chromatin structures. HDAC7, a member of the class II HDACs, is specifically expressed in the vascular endothelium and contributes to the maintenance of vascular integrity during early embryogenesis.^9^, ^10^, ^11^ Due to alternative splicing, there are 4 and 8 transcript variants in human and mouse *Hdac7* mRNA, respectively. In this study, we demonstrated that a sORF within a mouse *Hdac7* transcript variant could be translated, giving rise to a 7‐amino‐acid (7‐aa) peptide (7A). This peptide could act as a signal transducer through transferring a phosphate group between a kinase and a substrate; this action modulated stem cell antigen‐1‐positive VPC (Sca1^+^‐VPC) activation and its effects on vascular injury repair and angiogenesis in ischemic tissues.

## 2 MATERIALS AND METHODS

### 2.1 Materials

All cell culture media and serum were purchased from Thermo Fisher Scientific (Waltham, Massachusetts), whereas cell culture supplements and growth factors were purchased from Sigma (St. Louis, Missouri). The peptides of 7A (MHSPGADC), MEKK1 (SRRS[pSer]RIKAPSRNTC), and 14‐3‐3γ (KRA[pThr]VVESSEKAYSC) were synthesized and used to raise anti‐7A, anti‐pMEKK1Ser393, and anti‐p14‐3‐3γThr145 antibodies in rabbit by GenScript (Piscataway, New Jersey). The antibodies against CD31 (ab28364), Sca‐1 (ab51317), 14‐3‐3γ (ab115176), and MEKK1 (ab55653) were purchased from Abcam (Cambridge, UK). The antibodies against phospho‐Ser (P5872), phospho‐Thr (P3555), FLAG (F1804), and HA (H6908) were purchased from Sigma. The antibody against GAPDH (sc‐25 778), HDAC7(sc‐74 563), and the siRNAs (control siRNA [sc‐37007], MEKK1 siRNA [sc‐35899], and 14‐3‐3γ siRNA [sc‐29 584]) were purchased from Santa Cruz Biotechnology (Dallas, Texas). The antibodies against phosphohistidine (MABS1341, 1‐pHis clone SC50‐3; MABs1352, 3‐pHis clone SC56‐2) were purchased from Merck (Kenilworth, New Jersey). The antibody against pMKK4S257/T261 (ABS160) was from Millipore (Berlin, Germany), and antibody against MKK4 (9152 seconds) was from Cell Signaling Technology (Leiden, The Netherlands). All secondary antibodies were purchased from DAKO (Glostrup, Denmark). All other chemicals were purchased from Sigma. All peptides (see list in Figure S6) and DNA fragments were synthesized by GenScript.

### 2.2 Cell culture

Sca1^+^‐VPCs were isolated from the outgrowth of adventitial tissues of mouse arterial vessels, as previously described.^12^, ^13^ Briefly, the arterial vessels were harvested from C57BL/6J mice (Charles River, Margate, Kent, UK) or *Hd7‐7sFLAG* transgenic mice and cut into 2‐mm rings after the removal of the intima and media; the pieces were placed in gelatin‐coated flasks and incubated at 37°C in a humidified incubator supplemented with 5% CO_2_ for 6 hours. Stem cell culture medium ([Dulbecco's modified Eagle medium (DMEM); ATCC, Rockville, Maryland] supplemented with 10 ng/mL recombinant human leukemia inhibitory factor [Chemicon, Temecula, California], 10% fetal bovine serum [FBS, ATCC], 0.1 mmol/L 2‐mercaptoethanol, 100 U/mL penicillin, and 100 U/mL streptomycin) was added and refreshed every other day until the cells reached 80% confluence. The cells were expanded and subjected to Sca‐1^+^ cell purification using anti‐Sca‐1 immunomagnetic microbeads Miltenyi Biotec (Bergisch Gladbach, Germany). The purity of isolated Sca‐1^+^ cells was confirmed to around 85% using flow cytometry.^12^, ^13^, ^14^ The Sca‐1^+^‐VPCs were maintained in stem cell culture medium and split every other day. Cells passaged up to 30 times were used in this study, and Sca1‐selection was performed every five passages.

### 2.3 Fluorescence in situ hybridization

Sca1^+^‐VPCs were seeded at a density of 10^4^ cells/well on gelatin‐coated ϕ13mm coverslip in 24‐well plates in differentiation medium and incubated for 24 hours. The cells were cultured in alpha‐minimum essential medium (alpha‐MEM) supplemented without serum for 4 hours, then treated with 5 ng/mL vascular endothelial growth factor (VEGF; VEGF‐164, R&D systems, Minneapolis) for 30 minutes, followed by fixation with 4% paraformaldehyde/phosphate‐buffered saline (PBS) solution at room temperature for 15 minutes. Fifteen minutes prior to fixation, 10 μg/mL of puromycin was added. Same volume of 1% bovine serum albumin (BSA) and dimethyl sulfoxide (DMSO) were included as control for VEGF and puromycin, respectively. The fixed cells were washed three times with PBS at 5 minutes each, permeabilized with 0.2% Triton X‐100/PBS at room temperature for 30 minutes, followed by washing with PBS for three times at 5 minutes each. The cells were then prehybridized with 400 μL/well prehybridization buffer (6× saline sodium citrate [SSC]), 5% BSA, 100 μg/mL single‐stranded salmon sperm DNA, 10 mM EDTA) at 42°C water bath for 1 hour. After the removal of the prehybridization buffer, 400 μL/well of hybridization buffer (prehybridization buffer containing 0.4 μmol/L of the Alexa Fluor‐488/594‐labeled cDNA probes) was added and incubated at 42°C water bath for 1 hour, followed by washing with 1× SSC for 5 times at 5 minutes each. The nucleus was counterstained with DAPI for 5 minutes, followed by two washings with 1× SSC. The probe sequences were:5′ > AlexaFluor594‐ccgcgccggggctgtgcatccagggg<3′ and 5′>AlexaFluor488‐gaatgtcttagcaggctgtggggactcact<3′(Sigma). The coverslips were picked up and placed upside down on slides with fluorescence mounting medium (DAKO) and subjected to confocal microscope observation and image taken. Images were taken using an SP5 confocal microscope (Leica, Germany) and processed using Adobe Photoshop software. Magnification is indicated in figure legends as scale bars.

### 2.4 Immunofluorescence staining

Mouse tissues were harvested and immediately frozen with liquid nitrogen. The frozen tissues were then embedded in optical coherence tomography at −20°C, followed by cryosection with a thickness of 5–10 μm. The cells cultured on slides or the cryosections were fixed with 4% paraformaldehyde, permeabilized with 0.1% Triton X‐100 in PBS for 15 minutes, and blocked with 10% FBS/PBS for 1 hour. The primary antibodies were diluted in 10% FBS/PBS by the dilution factor recommended by the suppliers, applied to the cell samples and incubated at 37°C for 1.5 hours or at 4°C overnight. The secondary antibodies were diluted at 1:1000 in 10% FBS/PBS, then applied and incubated at 37°C for 45 minutes. The cell nucleus was counterstained with DAPI at room temperature for 5 minutes, and coverslips were mounted on slides with fluorescent mounting media. For 7A blocking experiments, the anti‐7A antibody was preincubated with 7A (1 μg Ab:1 μg peptide) overnight and then used as the same dilution factor as the nonblocking antibody.

### 2.5 Enzyme‐linked immunosorbent assay

For enzyme‐linked immunosorbent assay (ELISA) performed on cells, the undifferentiated or 3‐day differentiated Sca1^+^‐VPCs were seeded in 96‐well plates and treated as described in the figure legends. The cells were fixed with 4% paraformaldehyde at room temperature for 10 minutes, permeabilized with 0.1% Triton X‐100 in PBS for 15 minutes, treated with 3% H_2_O_2_ for 20 minutes to quench endogenous horseradish peroxidase (HRP), and blocked with 10% FBS/PBS for 1 hour, followed by incubation with primary antibodies at 37°C for 1 hour and HRP‐conjugated secondary antibodies at 37°C for 45 minutes. The associated HRP was revealed by incubation with phosphate citrate buffer (50 mmol/L phosphate‐citrate, pH 5.0, 0.03% sodium perborate) containing 0.17 mg/mL of *o*‐dianisidine dihydrochloride, and absorbance was measured at 405 nm by a Genios Pro‐Tecan microtiter plate reader (MTX Lab Systems, Inc., Virginia). For ELISA on peptides, 100 μL of peptides in PBS (0.1 mg/mL, 7Sp, 7Avp, 7Ap, b‐7Ap, and 7Ap‐b) were added to 96‐well microtiter plates in triplicate and incubated at 37°C overnight. PBS and VEGF (5 ng/mL)‐treated Sca1^+^‐VPCs cell lysate (100 μL at 0.1 mg/mL) were included as control. The plates were then fixed with 4% paraformaldehyde at room temperature for 10 minutes, treated with 3% H_2_O_2_ for 20 minutes to quench endogenous HRP, and blocked with 10% FBS/PBS for 1 hour, followed by similar procedure as described above. For ELISA on beads, the immunoprecipitation beads or biotin‐peptide pull‐down assay beads were incubated with primary and HRP‐conjugated secondary antibodies as described above, with A405 nm measured on a Bio‐Rad Smartpec Plus Spectrophotometer (Hercules, California).

### 2.6 Immunoprecipitation and biotin‐peptide pull‐down assay

The cells were lysed in lysis buffer (10 mmol/L Tris‐Cl pH 7.5, 120 mmol/L NaCl, 1 mmol/L EDTA pH 8.0, 1% Triton X‐100 plus protease inhibitors [Roche]) by rotation at 4°C for 1 hour. The protein concentration was measured with Biorad Protein Assay (BIO‐RAD, 5000006) by the protocol provided. *For the immunoprecipitation assay*, 1 mg cell lysate was incubated with 2 μg primary antibody or normal IgG and three volumes of Triton X‐100‐free lysis buffer on a rotator at 4°C for 2 hours. Then, 10 μL Protein G‐agarose beads (Sigma) was added, and the liquid was incubated for another 2 hours followed by PBS washing and ELISA assays. *For the biotin‐peptide pull‐down assay*, 5 μg biotin‐labeled peptide was incubated with 50 μg cell lysates and three volumes of Triton X‐100‐free lysis buffer at 37°C for 1 hour. Then, 10 μL streptavidin‐agarose beads (Sigma) was added, and the liquid was incubated for an additional hour followed by washing six times with lysis buffer containing 0.5% Triton X‐100. The beads were incubated with 1× sodium dodecyl sulfate (SDS) loading buffer (10 mmol/L Tris‐Cl pH 7.5, 120 mmol/L NaCl, 2% SDS, 5% glycerol, 0.025% bromophenol blue, 1% 2‐mercaptoethanol). The eluate was subjected to sodium dodecyl sulfate‐polyacrylamide gel electrophoresis (SDS‐PAGE) followed by Western blot or proteomics analysis (Computational Biology Research Group, Oxford University). The beads were washed six times with lysis buffer containing 1% SDS followed by the ELISA assay.

### 2.7 RNA extraction

Total RNA extraction from Sca‐1+ progenitor cells was performed by applying RNeasy Mini kit (QIAGEN Inc., 74 106). According to manufacturer's instruction, cells were washed three times by PBS, disrupted by proportional amount of RLT lysis buffer, and then scraped off from the 6‐well plate or T25 flasks. The lysate was transferred into a mini QIAshredder spin column and centrifuged at full speed for 2 minutes. Same volume of 70% ethanol was added to the lysate and the mixture was transferred to RNeasy mini column for a 35‐second centrifuge at full speed. The flow was discarded and 700 μL of RW1 was added to RNeasy mini column. After 30‐second centrifuge, the flow was discarded and 500 μL of RPE was added to the column twice for washing away the ethanol. The flow was then discarded and the column was centrifuged within a new collection tube for 2 minutes at full speed to ensure no solution outside the column. At last, the RNeasy mini column was transferred into a new 1.5 mL RNA‐free tube, and 35 μL diethypyrocarbonate (DEPC) water (Invitrogen) was added to the membrane of the column, followed by 1‐minute centrifuge at full speed. The RNA concentration was measured by a nanodrop spectrophotometer ND‐1000 (Thermo Fisher Scientific, UK) at the absorbance at 280 nm.

### 2.8 Reverse transcription

Reverse transcription (RT) was achieved by Quanti Tect Reverse Transcription Kit (Qiagen, 205 311), according to the manufacturer's instructions. Briefly, 1 μg of RNA template, 2 μL of gDNA wipeout buffer and enough volume of RNase‐free water were mixed in the tube with a total volume 14 μL. The tube was placed in a RT‐polymerase chain reaction (PCR) machine (TECHNE TC‐412, Bibby Scientific, UK) at 42°C for 2 minutes, after which 1 μL of RT enzyme, 1 μL of primer mix and 4 μL of RT buffer, making up to 6 μL of reaction volume, was added to the tube mentioned above. Then the mixture was incubated at 42°C for 15 minutes and subsequently 95°C for 3 minutes. The cDNA obtained was diluted into 100 μL by using DEPC‐treated water, acquiring a final concentration of 10 ng/μL.

### 2.9 Quantitative real‐time PCR

Quantitative real‐time PCR was performed by applying SYBR green system (Qiagen, 204 057). The target gene was amplified in a duplex in 20 μL PCR mixtures (10 μL Sybr Green, 2 μL cDNA template, 1.6 μL optimized primers, and 6.4 μL DEPC water) which was loaded into a 96‐well plate (Eppendorf White, Eppendorf, UK). The plate was centrifuged at 1000 rpm for 5 minutes before running the program in qPCR machine. Ct values were established using EPPENDORF Mastercycler ep realplex. GAPDH served as an endogenous control.

### 2.10 Western blot analysis

Cells with or without treatment were lysed using RIPA buffer (Life Tech, 89 901) with phosphatase inhibitor tablets (Roche, 04906845001) and protease inhibitors (Roche, 11 873 580 001). The lysate was sonicated using a Branson Sonifier 150 at level 1 for 6 seconds twice prior to 50‐minute incubation on ice. The lysate was then centrifuged at 15 000*g* for 12 minutes at 4°C. The supernatant was collected and transferred to a new 1.5 mL tube. The concentration of proteins was measured by performing Biorad Protein Assay (BIO‐RAD, 5000006). A 20 μg of lysate mixed with SDS loading buffer was loaded into a NuPage 4%‐12% Bis Tris‐gel immersed in NuPage MOPS SDS running buffer, followed by standard Western blot procedures.

### 2.11 In‐gel phosphorylation

The recombinant 14‐3‐3 γ protein (H00007532‐P01) was purchased from Novus Biologicals (Littleton, Colorado). One hundred nanograms of 14‐3‐3γ protein were diluted in 250 μL of 1× SDS loading buffer, and 25 μL per lane of the diluted 14‐3‐3γ protein was applied to SDS‐PAGE. One lane together with the protein marker was cut and subjected to silver staining (Thermo Fisher Scientific) by the protocol provided. The 14‐3‐3γ protein bands in the remaining gel were cut and incubated in Novex Zymogram Renaturing buffer (Thermo Fisher Scientific) at room temperature for 1 hour. After washing three times with tris‐buffer saline and tween 20 (TBST) buffer (25 mmol/L Tris‐Cl pH 7.5, 120 mmol/L NaCl, 1 mM EDTA pH 8.0, 0.1% Tween 20), the gel bands were incubated in an equal volume of TBST containing 2 ng/mL peptides at 37°C for 30 minutes. The bands were reorganized and transferred to Hybond polyvinylidene difluoride membrane (GE Health) followed by a standard Western blot procedure.

### 2.12 siRNA knockdown assay

The Sca1^+^‐VPCs were subcultured 1:3 in differentiation medium in fibronectin‐coated T25 flasks. Twenty‐four hours later, the cells were washed twice with and incubated in serum‐free DMEM containing 0.05 mmol/L 2‐mercaptoethanol for 1 hour. Ten microliters of 10 μmol/L siRNA/flask was introduced into the cells with Lipofectamine RNAiMax transfection reagent (Thermo Fisher Scientific) according to the protocol provided. The transfected cells were further cultured in differentiation medium for 72 hours followed by 5 ng/mL of VEGF treatment in serum‐free medium for 30 minutes and ELISA assays and Western blot analysis.

### 2.13 Transwell migration assay

Of note, 3 × 10^4^ Sca1+‐VPCs in 200 μL serum‐free DMEM were seeded in the insert of 24‐well transwell with 8 μm pore and placed in the 24‐well transwell plate with the holder containing 600 μL/well of DMEM plus 1 ng/mL peptide and/or 5 ng/mL VEGF. The whole set was placed in cell culture incubator and incubated for 6 hours, followed by staining with crystal violet. After removal of the cells inside the insert, the migrated cells on the outside membrane of the insert were observed under microscope. Images were taken and cell number was calculated with ImageJ. The experiments were performed in triplicate.

### 2.14 Image quantification

For the study of CD31+ and Sca1+ cells in skeletal muscle of ischemic legs, immunofluorescence images were obtained in a blinded manner from three different fields of each animal. With the ImageJ software, each image was converted into a binary image, and the integrated density (DInt) and area were calculated. Then, the values were corrected by the number of nuclei.

### 2.15 *Hd7‐7sFLAG* transgenic mice generation, femoral artery injury, and hind limb ischemia

All animal experiments were performed according to protocols approved by the Institutional Committee for Use and Care of Laboratory Animals, and all the procedures conformed to the guidelines from Directive 2010/63/EU of the European Parliament. A single dose of ketamine (75 mg/kg) and medetomidine HCl (1 mg/kg) were injected into peritoneal cavity. After surgery, the mice were kept in a warm incubator until they got awake.

The *Hd7‐7sFLAG* transgenic mice were generated by Cyagen Biosciences Inc. (Santa Clara, California), in which the 7A sequence (MHSPGAD, ATGCACAGCCCCGGCGCGGACTGA) was replaced by the scrabbled 7S sequence (MPHASGD) tagged with a FLAG sequence (MPHASGDYKDDDDKAD, ATGCCCCACGCGTCGGGCGACTACAAAGACGATGACGACAAGGCGGACTGA). This handling only affects the first 5‐aa within the 7A ORF without any change to the promoter or the exon splicing sites. Therefore, the transcription and splicing of HDAC7 mRNA should not be affected. This transgenic mouse line can serve as a knockout model for 7A.

The femoral artery injury model was introduced into WT and KO mice as previously described.^15^ The uninjured sham control was included. Six mice were used for each group. The injured vessels were harvested at days 3, 7, and 21 postsurgery and cryo‐sectioned, followed by immunofluorescence staining (days 3 and 7) or Hemotoxylin and Eosin (H&E) staining (day 21). The hind limb ischemia model was introduced into WT and KO mice as previously described.^16^ Briefly, the femoral artery in the left leg was banded and cut. Six mice were used for each group. The foot blood perfusion was scanned on both legs using the Doppler Scanner at 30 minutes, days 7 and 14 post‐surgery. The images were analyzed with the software moorLDI V6.0. The mice were sacrificed humanely at day 14, and the leg skeletal muscle tissues were harvested and cryosectioned, followed by immunofluorescence staining. For the rescue experiments in both types of mouse models, 200 μL of 25% Pluronic‐127 gel containing 10 ng/mL of 7A peptide was applied surrounding the injured vessels.

### 2.16 Statistical analysis

The data expressed as the mean ± standard error of the mean were analyzed using GraphPad Prism 7 with a *t* test for pairwise comparisons or analysis of variance, followed by multiple comparison tests for comparisons involving more than two groups, and significance is depicted by asterisks, *P < .05; **P < .01, ***P < .001. A value of *P* < .05 was considered to be significant.

### 2.17 Statement of animal studies

All animal experiments were performed according to the protocols approved by the Institutional Committee for Use and Care of Laboratory Animals under the UK Home Office Project License PPL70/7266.

## 3 RESULTS

### 3.1 The 7‐aa peptide could be translated in vitro and in vivo

**Figure 1:**
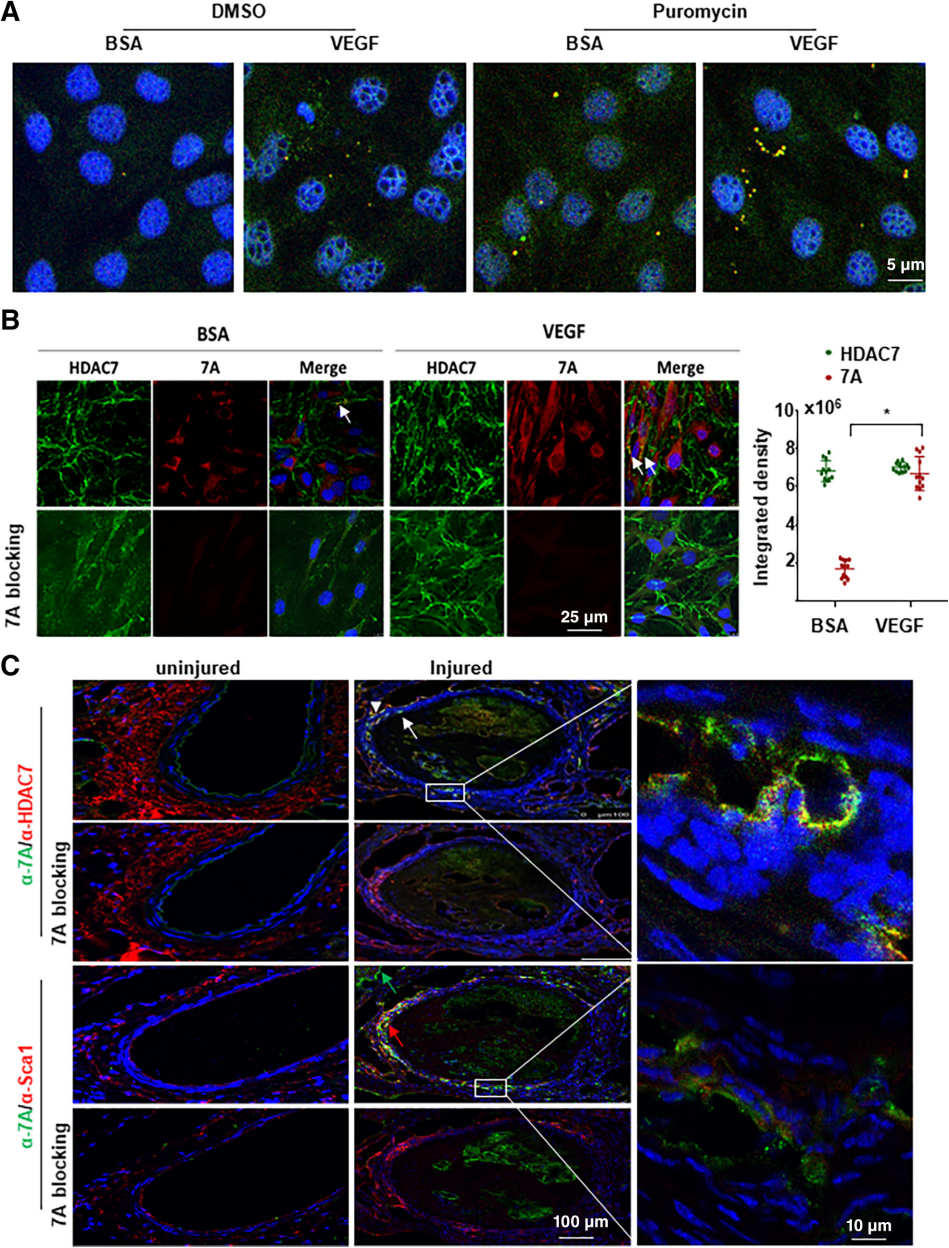
7A could be translated in vitro and in vivo. A, The 57‐bp intron containing *Hdac7* mRNA existed in the cytosol as revealed by fluorescence in situ hybridization with Alexa Fluor‐594 and Alexa Fluor‐488‐labeled oligo DNA probed complementary to the short open reading frame and intron sequences respectively. Sca1^+^‐VPCs were treated with 5 ng/mL vascular endothelial growth factor (VEGF) for 30 minutes in the absence or presence of 10 μg/mL puromycin (last 15 minutes), followed by hybridization. B, VEGF enhanced 7A expression in Sca1^+^‐VPCs. Sca1^+^‐VPCs were treated with 5 ng/mL VEGF for 30 minutes, followed by double immunofluorescence staining with anti‐7A (red) and anti‐HDAC7 (green) antibodies. 7A blocking indicates that the anti‐7A antibody was preincubated with synthetic 7A peptide (1:1) overnight. Data are presented as mean ± SD, n = 12 cell cultures per group from three individual preparations. For statistical analysis, the Student's *t* test was used. *P < .05. C, Vascular injury induced 7A expression in Sca1^+^ cells. Femoral artery injury was introduced into C57BL/6J mice (n = 6). The injured arteries were harvested at day 7 postsurgery, followed by immunofluorescence staining with anti‐7A (green) and HDAC7 (red) or Sca1 (red) antibodies. White arrowhead showed HDAC7, white arrow showed 7A, green arrow showed 7A^+^/Sca1^−^ cell, red arrow showed 7A^+^/Sca1^+^ cell. The data presented are representative images of three independent experiments or from six mice per group for in vivo experiments. VPC, vascular progenitor cell

In the 5′ untranslated region (UTR) of mouse *Hdac7* transcript variant 2 (*NM_001204276.1*), there is a sORF encoding a 7‐aa peptide (7A, MHSPGAD) with three cascade in‐frame stop codons. Our previous studies demonstrated that this *Hdac7* mRNA underwent further splicing to remove these stop codons, joining the sORF with the main ORF.^17^ These findings suggest that the ATG start codon in the sORF can initiate translation. As three in‐frame sequential stop codons exist in the sORF, we speculated that this sORF might be translated alone. To test this, we firstly performed a FISH (fluorescence in situ hybridization) experiment. The Sca1^+^‐VPCs were isolated from the adventitia of arterial vessels via Sca1^+^ cell sorting^13^, ^18^ with around 85% purity and treated with 5 ng/mL VEGF in the absence or presence of 10 μg/mL puromycin, followed by hybridization with Alexa Fluor 488‐ and 594‐labelled oligo DNA probes, which were complementary to the intron and the sORF sequences, respectively (Figure S1A). The two complementary sequences for these two probes are next to each other. If they bind to the same molecule, an overlapped fluorescence signal will appear. Indeed, we observed a few cells contained some spots with an overlapped fluorescence signal in the cytoplasm (Figure 1A and Figure S1B with arrow indication). VEGF could increase such spots number and positive cell number. These results suggest that the intron containing mRNA exists in the cytoplasm. Most importantly, the presence of puromycin significantly increased the number of the overlapped spots in the cytosol within single cell and the number of cells containing such spots (see images for puromycin vs DMSO in Figure 1A and Figure S1B). Puromycin can remove ribosome from the mRNA molecule,^19^ which makes the mRNA accessible to the probes. These results not only demonstrate the existence of the intron‐containing mRNA in the cytosol but also indicate the binding of ribosome to the sORF. We also noticed that the presence of puromycin increased red signal in the nucleus, reflecting that the transcription and splicing of the intron in the nucleus was also increased. To assess whether the binding of ribosomes to sORF led to the 7A peptide translation, we raised a specific antibody against this 7A peptide and performed double immunofluorescence staining together with anti‐HDAC7 antibody. If the sORF joins the main ORF, the fluorescence for 7A and HDAC7 will overlap. The nonoverlapped fluorescence will suggest the separate translation of the 7A peptide and HDAC7 protein. As expected, some overlapped signal was observed (arrows in Figure 1B) but the majority was non‐overlapped and both types of signals were enhanced by VEGF treatment (Figure 1B). The pre‐incubation of anti‐7A antibody with 7A peptide totally blocked 7A staining (Figure 1B, 7A blocking). These results suggest that the sORF can indeed be translated alone to produce the 7A peptide and that VEGF stimulation can enhance this process.

Considering the in vitro‐cultured Sca1^+^‐VPCs were already activated cells, we speculated whether the endogenous 7A peptide was expressed constitutively in Sca‐1^+^ cells or only under stimulation in vivo. To test this, we performed immunofluorescence staining on sections from injured and uninjured mice femoral arteries. As shown in Figure 1C, there was positive staining for both HDAC7 and Sca‐1 but not for 7A peptide in uninjured vessels. Neither HDAC7 nor 7A was detected in the media layer of the uninjured femoral artery. However, positive staining for 7A (white arrows in Figure 1C) peptide and HDAC7 (white arrowhead in Figure 1C) was detected in the activated and migrated Sca‐1^+^ cells (red arrow in Figure 1C) in the injured vessels. There were some 7A^+^/Sca‐1^−^ cells (green arrow in Figure 1C) in the outer layer, the identity of these cells needs further investigation. Although there was nonspecific signal from the blood clot in the lumen, the 7A staining on the vessel wall was totally blocked by the preincubation of the antibody with the synthetic 7A peptide. The costaining of 7A/HDAC7 and 7A/Sca‐1 suggests that 7A and HDAC7 are co‐translated in the activated Sca‐1^+^ cells. In some cells, the staining for 7A and HDAC7 overlapped, which may suggest there is a splicing event and the 7A peptide joins the main protein.^17^ Further experiments with mouse heart tissues revealed that both 7A and HDAC7 were expressed in endothelium, some of the signal were overlapped (Figure S2). The difference on 7A and HDAC7 expression between coronary artery and femoral artery may reflect the EC heterogeneity along the arterial.^20^ Different Hdac7 mRNA transcript variants or protein isoforms may be expressed in different part of the arterial tree.

Recently, we published some data on the role of 7A in EC differentiation and its contribution to tissue engineered vessel graft^21^ and protection in cardiac fibrosis via hydrogel implication.^22^ In this study, we focused on the demonstration of the translation of the 7A peptide and its function in signal transduction and vascular remodeling.

### 3.2 7A deficiency attenuated vascular injury repair and angiogenesis in ischemic tissues

To better understand the function of 7A in vivo, we created a transgenic mouse line, *Hd7‐7sFLAG*, in which the first 5 amino acid of the sORF (MHSPG of MHSPGAD) was replaced by a 7S sequence that was tagged with a FLAG sequence (MPHASGDYKDDDDKA) through homologous recombination (Figure S3A). The last three nucleotides within the exon 1 of *Hdac7* mRNA (*NM_001204276.1*) remained unchanged. Thus theoretically, the transcription and normal splicing of this transcript variant should not be affected. However, the replacement may affect its further splicing.^17^ The expression of FLAG can serve as an indicator for the endogenous expression of the 7A peptide. Indeed, FLAG‐positive Sca1^+^ cells were detected in the adventitia of the aorta in transgenic mice (Figure S3B). This mice line can therefore serve as the knockout mice for the 7‐aa peptide. As the *Hdac7* mRNA transcript variant will not be able to undergo further splicing, the mice line can also serve as a model to investigate the role of the 22 amino acid in the N‐terminal of HDAC7 protein. In this study, we just focused on the 7‐aa peptide. We assessed the transcription of *Hdac7* mRNA from P1 and P2 promoters in lung and spleen tissues by quantitative RT‐PCR. There was no difference in lung between the wild‐type and the 7A‐deficient mice [*Hd7‐7sFLAG* ^*+/+*^ (KO)], but KO mice had slightly higher *Hdac7* mRNA (from both P1 and P2 promoters) in spleen tissues (Figure S3C). Western blot analysis revealed that there was no significant difference between wide‐type and KO mice, although spleen tissues showed abundant HDAC7 protein (Figure S3D). As we have shown that 7A and HDAC7 were expressed differentially in different tissues or organs, it would be worthy to enrich ECs and VPCs from the different organs or tissues to assess *Hdac7* transcription from different promoters and their translation regulation in future studies.

Our recently published data have shown that 7A especially 7Ap could increase angiogenesis in ischemic tissues and that inclusion of 7Ap in tissue engineered vessel graft could increase Sca1^+^‐VPCs recruitment in the grafts.^21^ To further investigate whether the translation of 7A in Sca1^+^‐VPCs contributes to re‐endothelialization in vascular injury repair and angiogenesis, we generated femoral artery injury and hind limb ischemia models using WT and KO mice.

**Figure 2:**
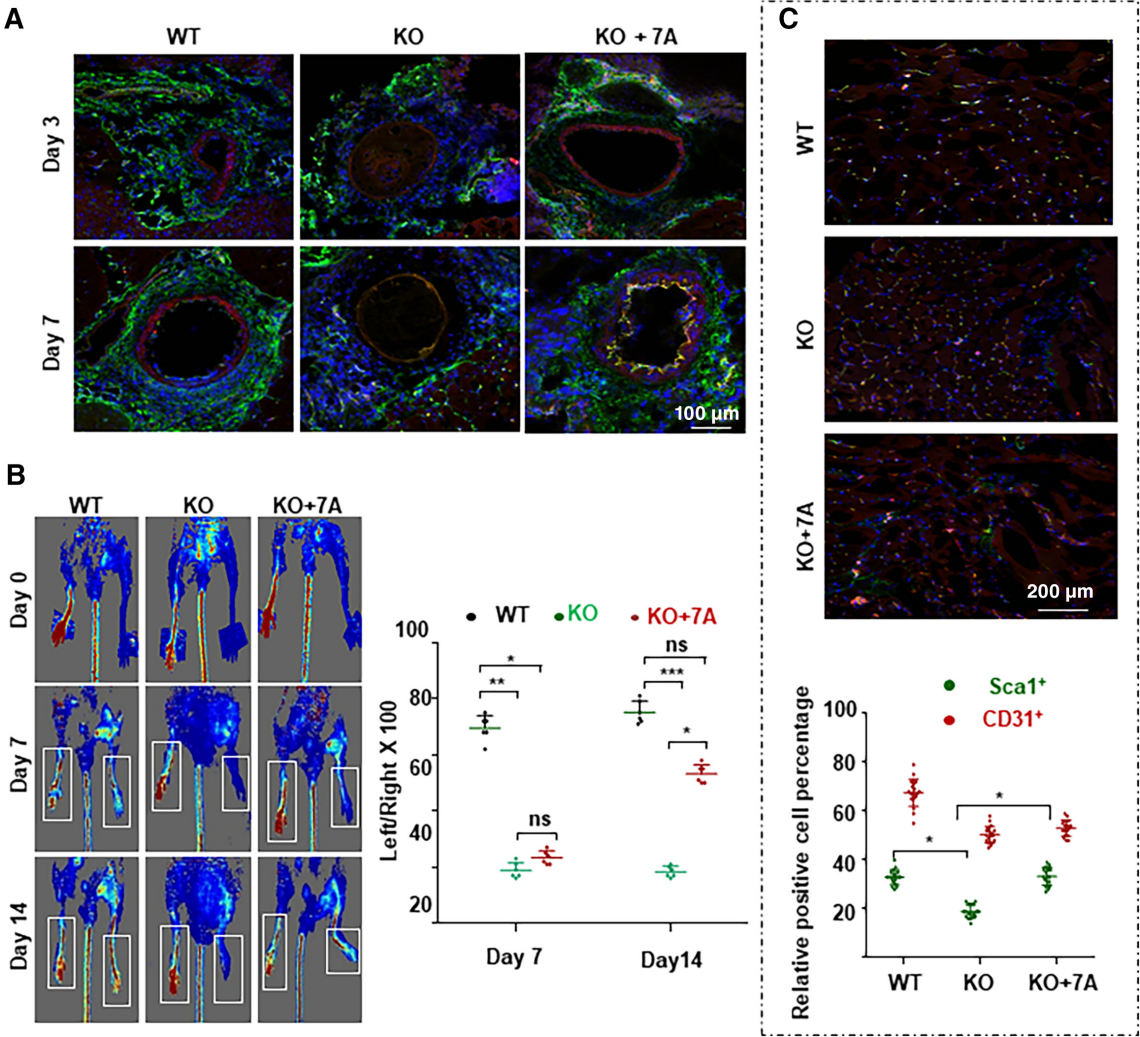
7A deficiency reduced re‐endothelialization in injured vessels and angiogenesis in ischemic tissues. A, 7A deficiency reduced re‐endothelialization in injured vessels, which was partly rescued by local administration of the 7A peptide. Femoral artery injury was introduced in WT or KO mice. Some KO mice received 200 μL of Pluronic F‐127 gel containing 10 ng/mL 7A peptide (KO + 7A). The injured vessels were harvested on days 3 and 7 postsurgery and subjected to cryo‐sectioning and double immunofluorescence staining with anti‐Sca1 (green) and anti‐CD31 (red) antibodies. The nuclei were counterstained with DAPI. B‐D, 7A deficiency attenuated the recovery of foot blood perfusion in the hind limb ischemia model, which was partly rescued by local administration of the 7A peptide. Hind limb ischemia was induced in WT or KO mice via femoral artery ligation, and some KO mice received 200 μL of Pluronic F‐127 gel containing 10 ng/mL 7A peptide (KO + 7A). Foot blood perfusion was measured by a Doppler Scanner on days 7 and 14 postsurgery (B, C). The mice were sacrificed on day 14, and Sca1^+^ (red) and CD31^+^ (green) cells in skeletal muscle tissue sections from the uninjured and injured legs were analyzed. The data presented are representative images or the mean values of six mice for each group. For statistical analysis, two‐way analysis of variance test followed by Tukey's multiple comparisons test was used. *P < .05

In the femoral artery injury model, many Sca1^+^‐VPCs could be observed on days 3 and 7 as well as obvious re‐endothelialization on day 7 in WT mice (Figure 2A, left). Considerably fewer Sca1^+^‐VPCs were observed in KO mice, and there was no obvious re‐endothelialization (Figure 2A, middle). Local administration of the 7A peptide via Pluronic‐F127 gel significantly improved re‐endothelialization (Figure 2A, right). The overlapped signal was observed in the endothelium of KO + 7A group at day 7 (Figure 2A right bottom), suggesting that 7A increased Sca1^+^‐VPCs differentiation into ECs and contribution to endothelium injury repair. These results suggest that the translation of 7A can contribute to Sca1^+^‐VPC activation and the re‐endothelialization of injured blood vessels. Vascular injury is usually linked to neointima formation. However, in this study, we did not observe obvious neointima formation in either WT or KO mice within 3 weeks (Figure S4). Local administration of the 7A peptide to KO mice slightly decreased the media thickness (Figure S4).

In the hind limb ischemia model, the KO mice showed a severe reduction in or loss of foot blood supply as revealed by Doppler scanner measurement (Figure 2B and C). Local delivery of the 7A peptide partly rescued this phenotype (KO + 7A in Figure 2B and C). Due to the poor blood supply, all KO mice developed moderate to severe foot necrosis, and two mice even lost the foot (Figure S5). All WT mice had mild necrosis on day 7 but recovered to the minimal level on day 14 (Figure S5). Local administration of the 7A peptide to KO mice significantly improved the necrosis to moderate or mild level (Figure S5). Immunofluorescence staining of the skeletal muscle tissues isolated from the injured leg with anti‐Sca1 and anti‐CD31 antibodies showed that there were considerably fewer Sca1^+^ cells in KO mice than in WT mice, and local administration of the 7A peptide significantly increased the number of Sca1^+^ cells (Figure 2C). There were slightly less CD31^+^ cells in KO mice (Figure 2C). These results indicate that the endogenous 7A peptide may participate in Sca1^+^‐VPC activation and affect its contribution to angiogenesis in ischemic tissue; furthermore, the exogenous 7A peptide may have therapeutic potential in the treatment of angiogenesis‐related diseases.

### 3.3 VEGF induced 7‐aa peptide phosphorylation via MEKK1

**Figure 3:**
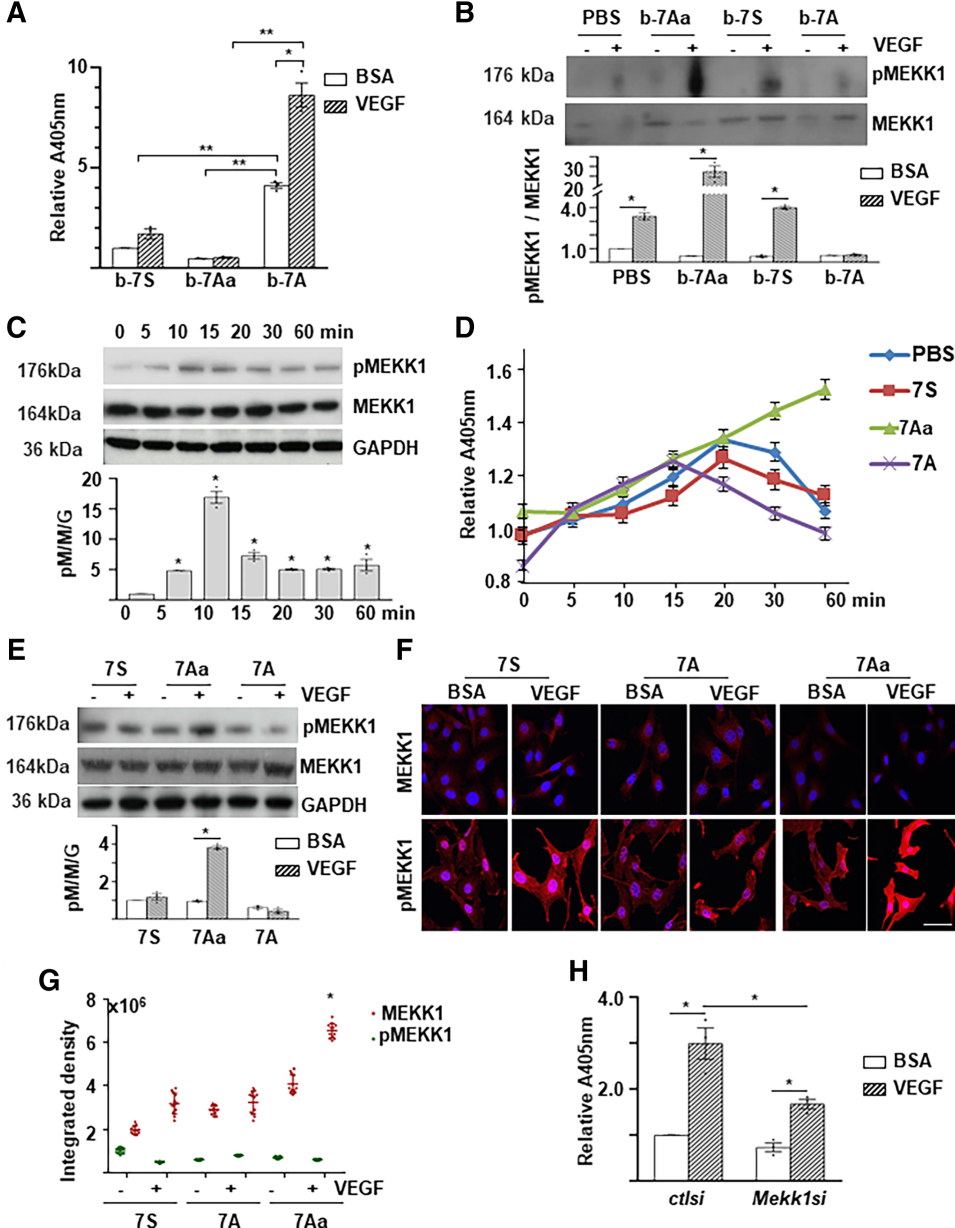
MEKK1 phosphorylated 7A upon vascular endothelial growth factor (VEGF) treatment. A, VEGF enhanced 7A phosphorylation. Biotin‐labeled peptides were incubated with cell lysates of VEGF‐treated/untreated Sca1^+^‐VPCs, followed by streptavidin beads pull‐down and enzyme‐linked immunosorbent assays (ELISAs) with anti‐phospho‐Ser antibody. B, The 7Aa peptide interacted physically with MEKK1. The bio‐peptide/protein complex was trapped by streptavidin beads and subjected to Western blot analysis. C, VEGF transiently activated MEKK1 phosphorylation. Sca1^+^‐VPCs (1 × 10^6^) were treated with 5 ng/mL VEGF in serum‐free medium for the indicated times and then subjected to Western blot analyses. D‐G, The 7Aa peptide retained MEKK1 phosphorylation. Sca1^+^‐VPCs were treated with 5 ng/mL VEGF in the presence of 1 ng/mL peptides for the indicated times and subjected to ELISA (D) or for 30 minutes and subjected to Western blot analyses (E) or immunofluorescence staining (F, Scale bar: 25 μm, G quantification). Data are presented as mean ± SD, n = 12 cell cultures per experimental group from three individual preparations. H, *Mekk1* knockdown attenuated VEGF‐mediated 7A phosphorylation. Sca1^+^‐VPCs(5 × 10^5^) were transfected with *Mekk1* siRNA (*Mekk1si*) and cultured for 3 days, followed by 5 ng/mL VEGF treatment for 30 minutes. Control siRNA (*ctlsi*) and 1% bovine serum albumin were included as controls. The cell lysates were subjected to bio‐peptide/streptavidin beads pull‐down arrays followed by ELISAs with an anti‐phospho‐Ser antibody. The data presented are representative images or the mean values of three independent experiments. For statistical analysis, the two‐way analysis of variance (ANOVA) followed by Tukey's multiple comparisons test was used in (A, B, E, G); the one‐way ANOVA followed by Holm‐Sidak's multiple comparisons test was used in (C, D); and the Student's *t* test was used in (H). *P < .05; **P < .01. 7A, 7‐aa peptide; 7Aa, 7‐aa peptide with a serine substituted with an alanine; 7S, scrabbled 7‐aa peptide; b‐7S/7A/7Aa, biotin‐labeled 7‐aa peptides. VPC, vascular progenitor cell

To investigate how 7A promote angiogenesis, we focused on 7A amino acid sequence, which contains an adjacent histidine and serine residue, the two of the four normally phosphorylated amino acids in eukaryotic proteins. Therefore, we wondered whether there was a phosphorylation event within the 7A peptide. To test this, we performed a special biotin‐labeled peptide pull‐down assay (Figure S6A). The biotin‐labeled peptides were incubated with VPC lysates and pulled down with streptavidin magnetic beads. ELISA with an anti‐phospho‐Ser antibody detected a positive signal in the bio‐7A/streptavidin beads, which was enhanced by VEGF treatment (Figure 3A); these findings demonstrate that 7A can be phosphorylated. This phosphorylation might be sequence‐specific, as the serine residue in the scrabbled 7A peptide (7S, MPHASGD) was not phosphorylated (Figure 3A). As expected, the substitution of serine with alanine (7Aa, MHAPGAD) totally abolished 7A phosphorylation (Figure 3A). All 7‐aa peptides used in this study were listed in Figure S6B.

To identify the potential upstream kinases for 7A phosphorylation, we performed a pilot proteomics analysis of peptide‐associated proteins using the special peptide/protein pulldown assay as described above to search for phosphorylation changes in response to VEGF treatment. In these assays, the biotin‐labeled 7Aa was included. As Ala substitution for Ser blocks the phosphate transfer from kinase to 7A, it may have more chance to detect phosphor‐peptides in bio‐7Aa associated kinases. We identified approximately 40 kinases associated with some of the biotin‐labeled 7‐aa peptides (see Figure S7A). The number of the total and the phosphorylated peptides were listed for the mitogen‐activated protein kinases (see Figure S7B). Indeed, a phosphorylated peptide (RSSRIK) from MEKK1 was detected in the bio‐7Aa samples. Therefore, MEKK1 was considered as a potential upstream kinase. To verify this, we raised a specific anti‐phospho‐MEKK1S393 antibody with the peptide SRRS (pSER)RIKAPSRNTC. With this antibody, we confirmed the association of bio‐7Aa and phosphorylated MEKK1 upon VEGF treatment (Figure 3B). Interestingly, b‐7S was also associated with MEKK1 (Figure 3B). The 7S sequence actually existed in some other proteins. The binding of 7S to MEKK1 might reflect the association of MEKK1 with 7S‐containing proteins, which may provide some clues to identify MEKK1 target proteins. Further experiments revealed that VEGF induced a transient increase in MEKK1S393 phosphorylation, which peaked at 10 minutes posttreatment (Figure 3C). Interestingly, the presence of 7A slightly decreased MEKK1 phosphorylation, whereas the addition of 7Aa maintained MEKK1 phosphorylation, as revealed by ELISA (Figure 3D), Western blot (Figure 3E), and immunofluorescence staining (Figure 3F&3G). To further confirm the involvement of MEKK1 in 7A phosphorylation, siRNA‐mediated *Mekk1* knockdown was performed. As shown in Figure 3H, *Mekk*1 knockdown significantly attenuated VEGF‐induced 7A phosphorylation. These results suggest that MEKK1 may function as an upstream kinase for 7A phosphorylation.

### 3.4 The phosphorylated 7A facilitated 14‐3‐3γ protein phosphorylation

**Figure 4:**
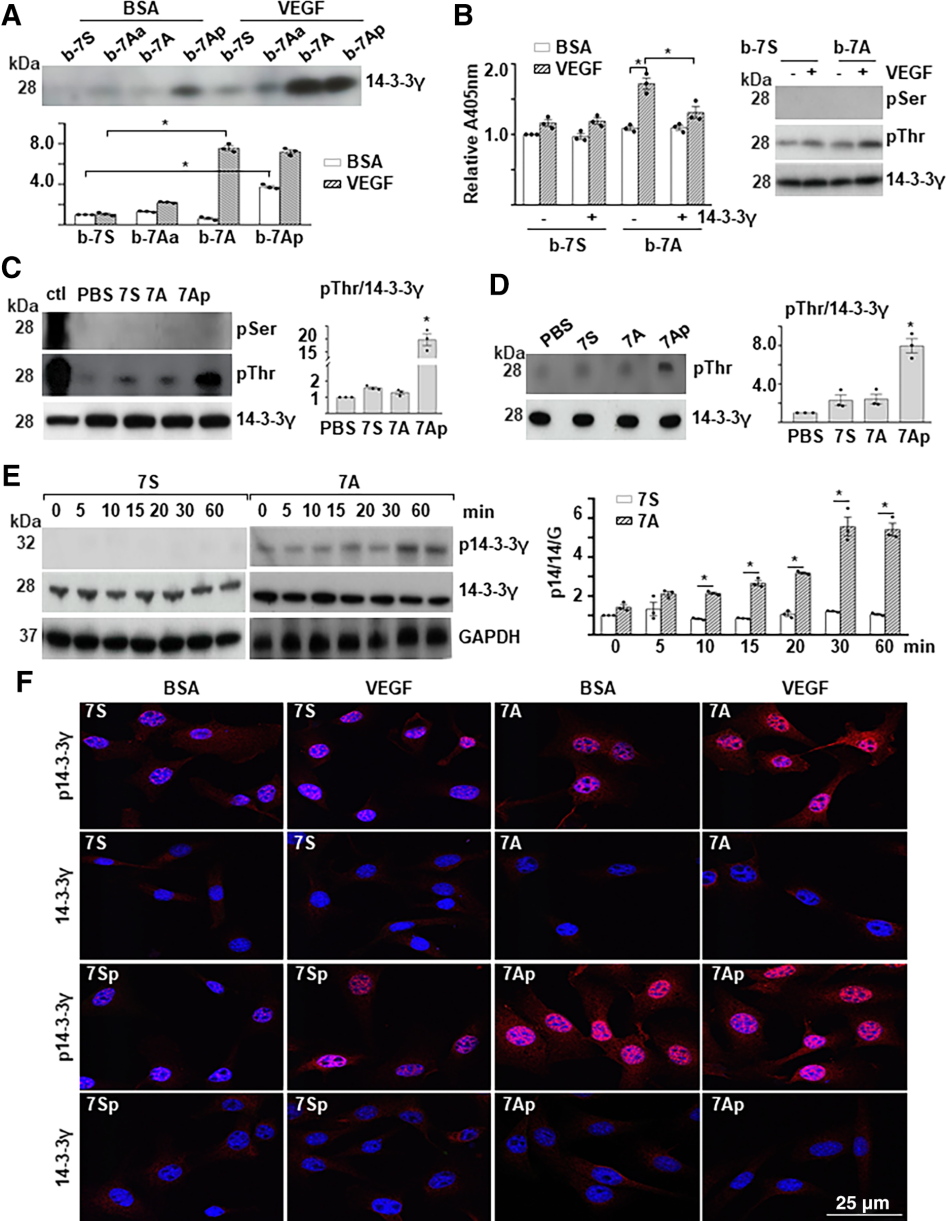
Phosphorylated 7A transferred the phosphate group to 14‐3‐3γT145. A, Phosphorylated 7A physically interacted with 14‐3‐3γ. The bio‐peptide/protein complex was trapped by streptavidin beads and subjected to Western blot analysis with anti‐14‐3‐3γ antibody. B, The peptide 7A mediated 14‐3‐3γ phosphorylation at threonine residues. Biotin‐labeled peptides/streptavidin beads were incubated with cell lysate from vascular endothelial growth factor (VEGF)‐treated Sca1^+^‐VPCs. After being washed with sodium dodecyl sulfate (SDS) solution, the beads were incubated with 10 ng of 14‐3‐3γ and eluted with SDS solution. The beads were subjected to enzyme‐linked immunosorbent assay with anti‐phospho‐Ser (pSer) antibody (Left), whereas the eluate was subjected to Western blotting (right). C, The synthetic 7Ap phosphorylated 14‐3‐3γ. The 14‐3‐3γ protein (10 ng) was incubated with peptides (1 ng) in a cell‐free buffer system followed by Western blotting. Fifty micrograms VEGF‐treated Sca1^+^‐VPCs lysate was included as a positive control (ctl). D, The 7Ap peptide phosphorylated 14‐3‐3γ in gel. The 14‐3‐3γ bands were cut from the sodium dodecyl sulfate‐polyacrylamide gel electrophoresis gel, renatured and incubated with peptides. The gel sections were rearranged and transferred to a polyvinylidene difluoride membrane followed by Western blot analysis. E, The 7A peptide enhanced VEGF‐mediated 14‐3‐3γ phosphorylation. Sca1^+^‐VPCs(1 × 10^6^) were pretreated with 1 ng/mL 7S or 7A for 1 hour and then treated with 5 ng/mL VEGF in the presence of the peptides for the time indicated followed by Western blot analysis. F, The 7Ap peptide induced 14‐3‐3γ phosphorylation and nuclear translocation. The Sca1^+^‐VPCs(1 × 10^6^) were pretreated with 1 ng/mL 7S or 7Sp (phosphorylated 7S) or 7A or 7Ap for 1 hour, treated with 5 ng/mL VEGF in the presence of the peptides for 30 minutes, and subject to immunofluorescence staining with anti‐phospho‐14‐3‐3γThr145 (p14‐3‐3γ) and anti‐14‐3‐3γ antibodies. Quantification was presented in Figure S4. Phosphate‐buffered saline and bovine serum albumin were included as control for peptides and VEGF, respectively. Scale bar: 25 μm. The data presented are representative images or mean values of three independent experiments. For statistical analysis, the two‐way analysis of variance (ANOVA) followed by Tukey's multiple comparisons test was used in (A) and (B); the one‐way ANOVA followed by Holm‐Sidak's multiple comparisons test was used in (C) and (D); Student's *t* test was used in (E). *P < .05. VPC, vascular progenitor cell

The 14‐3‐3 proteins are a family of conserved regulatory molecules ubiquitously expressed in all eukaryotic cells. Our previous study reported that HDAC7 could regulate cell growth by directly binding to 14‐3‐3 proteins.^23^ From the pilot proteomics study, we noticed that several components of the E‐cadherin complex and the 14‐3‐3 protein family were associated with the 7‐aa peptides (Figure S8A, B). Interestingly, we did not detect peptides from HDAC7 protein. One phosphorylated peptide (RATVVESSEK) from the 14‐3‐3γ protein and two phosphorylated peptides from the p120 protein (*CTNND1*) were detected in the bio‐7Ap (MH[pSer[PGAD, which had a synthetically phosphorylated serine residue) and VEGF‐treated bio‐7A samples (Figure S8C). These two proteins might be 7Ap target. In this study, we focused on 14‐3‐3γ. The physical binding of bio‐7A and bio‐7Ap to the 14‐3‐3γ protein was confirmed by Western blot analysis (Figure 4A). To test whether 7A was directly involved in 14‐3‐3γ phosphorylation, the phosphorylated bio‐7A peptide was purified by pull‐down assays and incubated with the commercially available recombinant 14‐3‐3γ protein; then, the phosphorylated 7A was detected by ELISA using the anti‐phospho‐Ser antibody, and the phosphorylated 14‐3‐3γ was detected with Western blot using the anti‐phospho‐Ser and anti‐phospho‐Thr antibodies. The phosphorylation signal in bio‐7A was increased by VEGF but was significantly reduced by further incubation with recombinant 14‐3‐3γ (Figure 4B, left). No phosphor‐Ser bands were detected, but a phosphor‐Thr‐positive band, which corresponded to the 14‐3‐3γ protein, was detected instead (Figure 4B, right). The direct incubation of synthetic 7Ap with the recombinant 14‐3‐3γ protein in a buffer system increases the threonine phosphorylation of the 14‐3‐3γ protein (Figure 4C).

As the commercial recombinant 14‐3‐3γ protein might contain trace amounts of other proteins that might facilitate the phosphorylation process, recombinant 14‐3‐3γ protein was subjected to SDS‐PAGE. The 14‐3‐3γ band was cut, followed by an in‐gel phosphorylation reaction. As shown in Figure 4D, 7Ap increased the phosphorylation of 14‐3‐3γ at a threonine residue. These results suggest that the threonine residue but not the serine residue within the peptide (RATVVESSEK) is phosphorylated and that 7Ap can facilitate the phosphorylation of the threonine residue (Thr145) in 14‐3‐3γ. Thereafter, we raised a specific antibody against phosphor‐14‐3‐3γThr145 (p14‐3‐3γ) with the KRA(pThr)VVESSEKAYSC peptide. With this antibody, we found that VEGF could increase the 7A‐induced phosphorylation of 14‐3‐3γ (Figure 4E). Further immunofluorescence staining revealed that phosphorylated 14‐3‐3γ was located mainly in the nucleus and upregulated by VEGF treatment, which was significantly enhanced by the presence of 7A (Figure 4F and Figure S9). Even more interesting, we found that synthetic phosphor‐7A (7Ap) increased 14‐3‐3γ phosphorylation in the absence of VEGF treatment (Figure 4F).

### 3.5 Adjacent histidine and proline residues were essential for the phosphorylation transfer

**Figure 5:**
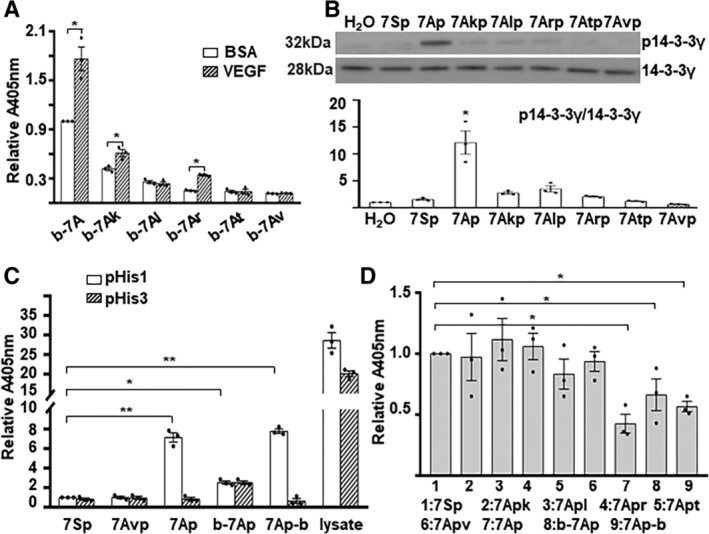
Histidine and proline were essential for the phosphate transfer. A, The alkaline and proline residues flanking the serine residue were essential for phosphorylation upon vascular endothelial growth factor (VEGF) treatment. Biotin‐labeled peptides were incubated with cell lysates from VEGF‐treated/untreated vascular progenitor cells (VPCs) and subjected to streptavidin‐agarose bead pulldown assays and enzyme‐linked immunosorbent assays (ELISAs) with an anti‐phospho‐Ser antibody. B, Both histidine and proline were critical for phosphorylating 14‐3‐3γ. 14‐3‐3γ (10 ng) was incubated with phosphorylated peptides (1 ng) in a cell‐free buffer system and subjected to Western blots with anti‐phospho‐14‐3‐3γThr145 (p14‐3‐3γ) and anti‐14‐3‐3γ antibodies. C, D, The phosphate group could be transferred from serine to histidine intramolecularly. Phosphorylated peptides (10 μg) were coated onto microtiter plates and subjected to ELISAs with anti‐phospho‐His (1‐pHis and 3‐pHis) (C) and phospho‐Ser (D) antibodies. In addition, 50 μg of VEGF‐treated VPC cell lysate was included as a positive control for phosphor‐histidine. The data presented are representative images or the mean of three values of independent experiments. For statistical analysis, Student's *t* test was used in (A) and the one‐way analysis of variance followed by Holm‐Sidak's multiple comparisons test was used in (B)‐(D). *P < .05; **P < .01

To investigate whether the two adjacent amino acid residues, histidine and proline, were involved in the phosphorylation transfer, we synthesized a set of mutant 7A peptides (Figure S6B). Peptide pull‐down assays were performed with biotin‐labeled 7A or mutant peptides (Figure S6A) and VEGF‐treated/untreated VPC cell lysates. As shown in Figure 5A, phosphorylated serine could be detected in b‐7Ak and b‐7Ar upon VEGF treatment, although it was attenuated compared with b‐7A. There was no phospho‐Ser signal in the other three mutants. These results suggest that an alkaline residue (arginine, histidine, or lysine) on the left side and a proline on the right side are essential for the receipt of the phosphate group to the serine residue in the 7A peptide. To further assess whether these substitutions affected the 7Ap to facilitate the target protein phosphorylation, recombinant 14‐3‐3γ was incubated with the synthetic phosphorylated mutant 7A peptides, and 14‐3‐3γ phosphorylation was determined by Western blot analysis. As shown in Figure 5B, all the substitutions lost the ability to facilitate 14‐3‐3γ phosphorylation, suggesting that histidine and proline are essential for facilitating phosphorylation.

Histidine is one of the four amino acids that can be phosphorylated at the N1 and/or N3 site in its imidazole ring in both bacterium and eucaryon.^24^, ^25^ As described above, the histidine residue was not essential for phosphorylation at the serine residue in the 7A peptide but was critical for the phosphorylation of 14‐3‐3γ; thus, we wondered whether there was an intramolecular phosphorylation transfer between the histidine and serine residues within the 7Ap peptide. Indeed, phosphorylated histidine at the N1 but not N3 position could be detected in synthetic 7Ap but not in 7SP or 7Avp, as revealed by micro‐titer plate‐coating/ELISA assays (Figure 5C). As expected, the phospho‐Ser signal was significantly lower in 7Ap compared with other mutants or 7Sp (Figure 5D). As the N‐terminal labeling of 7A with biotin might block the access of the antibody to the phosphorylated histidine, we synthesized a C‐terminal biotin‐labeled 7Ap‐b instead. Indeed, b‐7Ap showed a negligible phosphor‐His signal, but 7Ap‐b had a histidine phosphorylation signal similar to that of 7Ap (Figure 5C). The intramolecular phosphorylation transfer may facilitate the phosphate group transfer from 7Ap to the 14‐3‐3γ protein.

### 3.6 MEKK1, 7A, and 14‐3‐3γ formed a signaling pathway that contributed to Sca1^+^‐VPC migration

**Figure 6:**
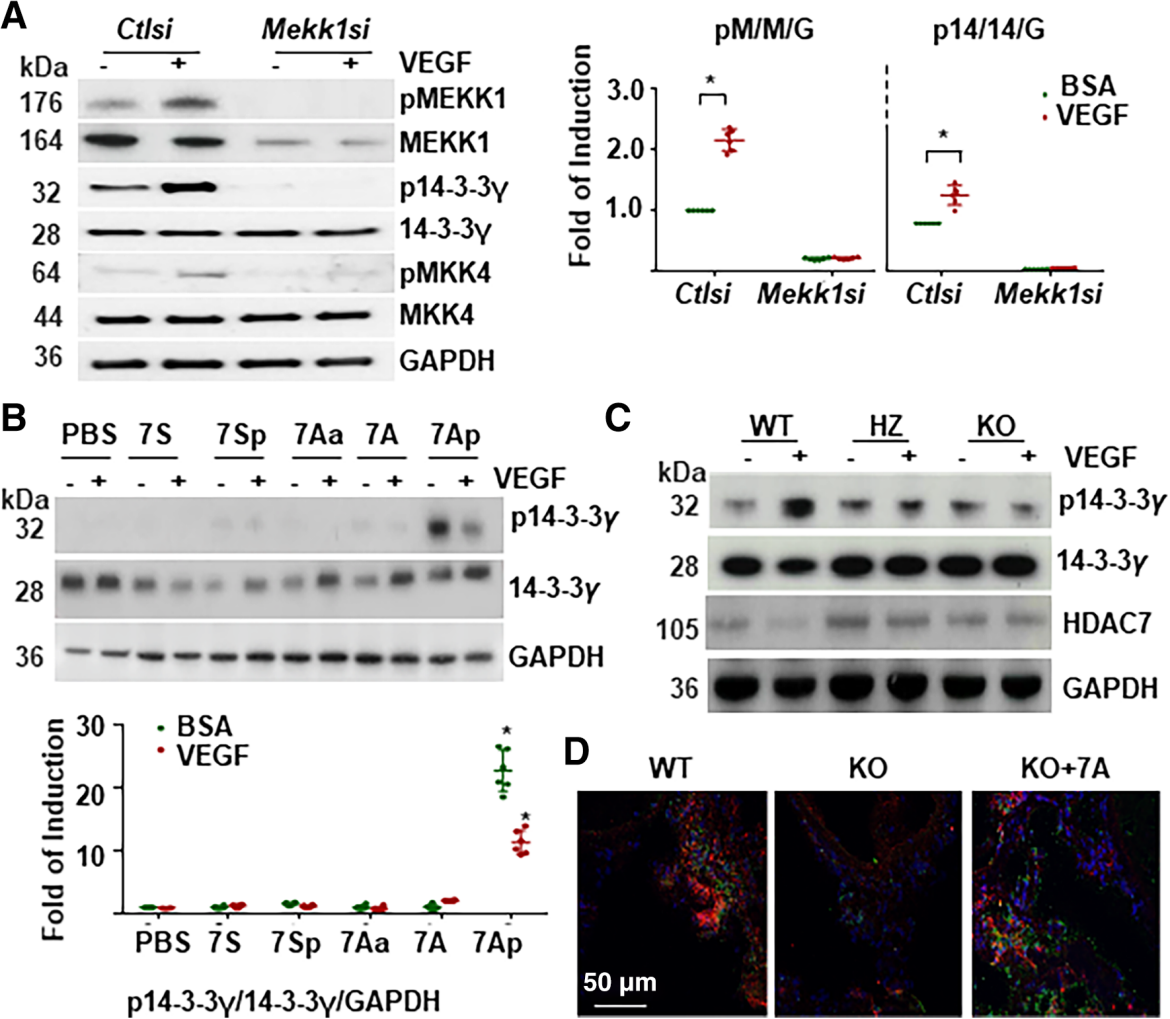
The MEKK1, 7A, and 14‐3‐3γ formed a novel signaling pathway downstream vascular endothelial growth factor (VEGF). A, Knockdown of *Mekk1* abolished VEGF‐induced 14‐3‐3γ phosphorylation in differentiated Sca1^+^‐VPCs. The Sca1^+^‐VPCs were transfected with *Mekk1* siRNA (*Mekk1si*) and cultured for 3 days, then treated with 5 ng/mL of VEGF in serum‐free medium for 30 minutes, followed by Western blotting with anti‐phospho‐MEKK1Ser393 (pMEKK1), anti‐MEKK1, anti‐phospho‐14‐3‐3γThr145 (p14‐3‐3γ), and anti‐14‐3‐3γ antibodies. Control siRNA (*ctlsi*) and GAPDH were included as siRNA and loading controls. pM/M/G: pMEKK1/MEKK1/ GAPDH; p14/14/G: p14‐3‐3γ/14‐3‐3γ/GAPDH. B, The 7Ap‐peptide induced 14‐3‐3γ phosphorylation independent of MEKK1. The *Mekk1* knockdown Sca1^+^‐VPCs were pretreated with 1 ng/mL peptides for 1 hour and then treated with 5 ng/mL VEGF for 30 minutes, followed by Western blotting with anti‐phospho‐14‐3‐3γThr145(p14‐3‐3γ) and anti‐14‐3‐3γ antibodies. Phosphate‐buffered saline and 1% bovine serum albumin were included as vehicle controls for peptide and VEGF, respectively. GAPDH was included as a loading control. C, 7A deficiency abolished VEGF‐induced 14‐3‐3γ phosphorylation. The Sca1^+^‐VPCs were isolated from wild‐type (WT), heterozygous (HZ), and homozygous (KO) mice arteries and treated with 5 ng/mL VEGF for 30 minutes, followed by Western blot analysis of 14‐3‐3γ phosphorylation. 14‐3‐3γ and GAPDH were included as loading controls. Note that HDAC7 expression was not affected by the transgenic handling. D, Local delivery of synthetic 7A‐peptide rescued 14‐3‐3γ phosphorylation in KO mice. Double immunofluorescence staining with anti‐Sca1 (red) and anti‐p14‐3‐3γT145 (green) antibodies was performed on the sections isolated from the femoral artery injured WT and KO mice 3 days postsurgery. In KO + 7A group, 200 μL of Pluronic F‐127 gel containing 10 ng/mL 7A was applied to the injury sites. Five mice were used for each group. The data presented are representative images or mean values of three independent experiments. For statistical analysis, the two‐way analysis of variance followed by Tukey's multiple comparisons test was used *P < .05. VPC, vascular progenitor cell

Having verified that 7A receives a phosphate group from the activated MEKK1 and in turn facilitates 14‐3‐3γ phosphorylation, we assessed whether 7A acted as a bridge between MEKK1 and 14‐3‐3γ. As shown in Figure 6A, the knockdown of *Mekk1* by siRNA abolished VEGF‐induced 14‐3‐3γThr145 phosphorylation and MKK4Ser257/Thr261 phosphorylation,^26^ indicating that MEKK1 is an upstream kinase for 14‐3‐3γ phosphorylation. In *Mekk1* knockdown Sca1^+^‐VPCs, the addition of 7Ap alone induced 14‐3‐3γThr145 phosphorylation, although VEGF treatment reduced 7Ap‐mediated 14‐3‐3γThr145 phosphorylation (Figure 6B). The Sca1^+^‐VPCs were isolated from WT, *Hd7‐7sFLAG* ^*+/−*^ (HZ), and *Hd7‐7sFLAG* ^*+/+*^ (KO) mice, treated with VEGF and subjected to Western blot analysis for 14‐3‐3γ phosphorylation. As shown in Figure 6C, VEGF induced 14‐3‐3γ phosphorylation in WT Sca1^+^‐VPCs, but the effect was significantly attenuated in HZ Sca1^+^‐VPCs and abolished in KO Sca1^+^‐VPCs. There was no difference on the basal level of HDAC7 protein between Sca1^+^‐VPCs from WT and KO mice. There seemed higher HDAC7 proteins in Sca1^+^‐VPCs from HZ mice. VEGF treatment decreased the HDAC7 protein level in Sca1^+^‐VPCs from WT and HZ mice but not in those from KO mice (Figure 6C). Further experiments detected fewer phosphor‐14‐3‐3γT145 positive cells in the vessel wall of KO mice compared with WT mice, which were subjected to femoral artery injury and sacrificed at day 3 postsurgery (Figure 6D). Local delivery of synthetic 7A peptide via Pluronic F‐127 gel significantly increased phosphor‐14‐3‐3γT145 positive cells in KO mice, even more than that in WT mice (Figure 6D). These results confirm that MEKK1, 7A, and 14‐3‐3γ form a signaling pathway downstream of VEGF treatment.

**Figure 7:**
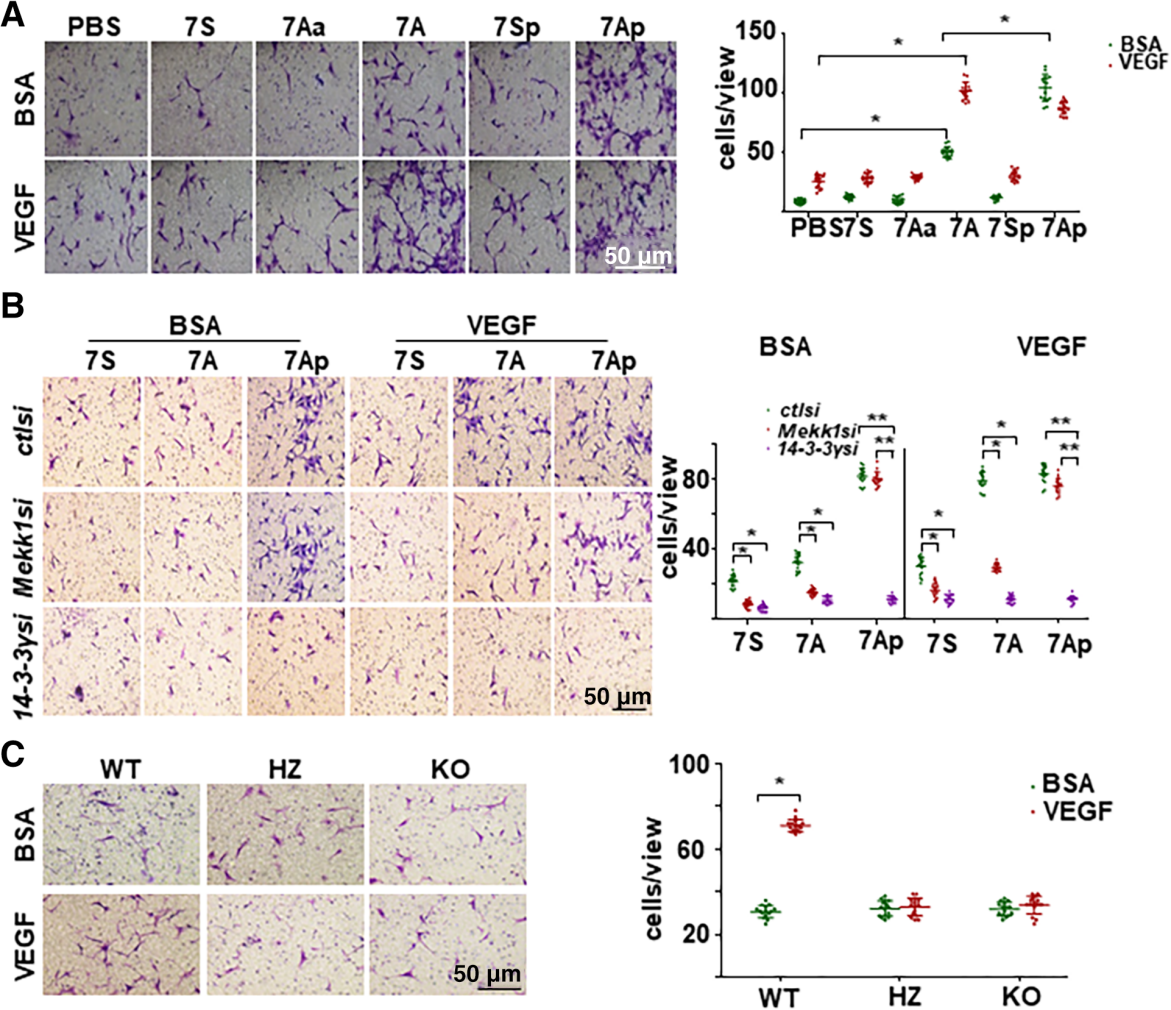
The MEKK1‐7A‐14‐3‐3γ pathway contributed to vascular progenitor cell (VPC) migration. A, 7A enhanced vascular endothelial growth factor (VEGF)‐induced VPC (5 × 10^4^) migration as revealed by transwell migration assays. Scale bar: 100 μm. B, *Mekk1* and *14‐3‐3γ* knockdown abolished VEGF‐mediated VPC migration. Transwell migration assays were performed using VPC (5 × 10^4^) transfected with control siRNA (ctlsi), *Mekk1 siRNA* (*Mekk1si*), and *14‐3‐3γ siRNA* (*14‐3‐3γsi*) and treated with 1 ng/mL peptides and 5 ng/mL VEGF. C, 7A deficiency abolished VEGF‐induced migration. Transwell migration assays were performed on VPCs isolated from wild‐type (WT), heterozygous (HZ), and homozygous (KO) mouse siblings. The left panels show crystal violet staining images, whereas the right panels show the mean values of the migrated cells for each image. Data are presented as mean ± SD, n = 15 cell cultures from six mice per experimental group. The data presented are representative images or as mean ± SD, n = 15 cell cultures per group of three independent experiments. For statistical analysis, two‐way analysis of variance followed by Tukey's multiple comparisons test was used in (A) and (B) and the Student's *t* test was used in (C). *P < .05; **P < .01

It is well established that local resident stem/progenitor cells contribute to vascular injury repair or disease development, which involves the mobilization of stem/progenitor cells from the stem cell niche and differentiation toward vascular lineages.^14^, ^27^ To test the cellular function of the 7A‐peptide, transwell migration assays were implicated to explore the effect of 7A on Sca1^+^‐VPC mobilization. As shown in Figure 7A, 7A increased Sca1^+^‐VPC migration and dramatically enhanced VEGF‐induced Sca1^+^‐VPC migration, whereas 7Ap alone was as effective as VEGF treatment. Interestingly, VEGF treatment attenuated 7Ap‐mediated VPC migration similar to 7Ap‐mediated 14‐3‐3γ phosphorylation. The knockdown of *Mekk1* ablated VEGF‐ and 7A‐ but not 7Ap‐induced Sca1^+^‐VPC migration (Figure 7B). However, the knockdown of 14‐3‐3γ abolished the Sca1^+^‐VPC migration induced by all factors (Figure 7B). Further experiments with Sca1^+^‐VPCs isolated from *Hd7‐7sFLAG* transgenic mice revealed that 7A deficiency abolished VEGF‐induced migration (Figure 7C). Therefore, these results demonstrate that the MEKK1/7A/14‐3‐3γ pathway is responsible for VEGF‐induced cell migration in Sca1^+^‐VPCs.

## 4 DISCUSSION

Recent studies indicate that one mRNA molecule can actually encode several peptides through different strategies including multiple ORFs or non‐AUG codon‐initiated translation, ribosomal frame shifting, stop codon read through, and termination‐dependent reinitiation.^28^, ^29^, ^30^, ^31^ Hundreds of putative coding sORFs have been identified by computing, proteomics, and ribosomal profiling, some of which have been confirmed.^32^, ^33^ Recently, Anderson et al reported that a functional 5KDa small peptide, myoregulin, could be translated from a putative long noncoding RNA.^34^ In this study, we demonstrate that a sORF within the 5′‐UTR of mouse *Hdac7* transcript variant 2 can be translated to produce a 7‐aa peptide.

Our previous study has demonstrated that the *Hdac7* transcript variant 2 could undergo further splicing to remove 57 nucleotides intron, joining the sORF with main ORF.^17^ In this study, the FISH experiments demonstrated the existence of the intron‐containing mRNA in the cytosol. Most importantly, the puromycin‐induced signal enhancement strongly suggests that ribosome binds to the sORF. The binding of the ribosomes may lead to the translation of the sORF alone as there are three cascade in‐frame stop codons. The direct evidence for the translation of the sORF is derived from the observation on the non‐overlapping of 7A and HDAC7 double immunofluorescence staining. If the 7A sequence exists in the N‐terminal of HDAC7 protein, the double staining should give an overlapped pattern due to the proximity that forms the basis of investigation on the protein‐protein interaction. Although we did observe overlapped staining of 7A and HDAC7 in vitro and in vivo, the non‐overlapped staining obviously existed, indicating that the sORF and mORF can be translated separately. The separate translation of these ORFs may provide a novel mechanism to regulate HDAC7 protein level, that is, the translation shift, in addition to the transcriptional suppression or posttranslational degradation by protein kinase D‐mediated phosphorylation.^35^, ^36^ The translation of the sORF and main ORF may be regulated separately. The binding of ribosome to the sORF may be mediated by the classic G‐N7‐cap structure.^37^ The three in‐frame sequential stop codons may guarantee the translation of the 7‐aa peptide stop effectively, avoiding the stop‐codon read through or reinitiation.^31^, ^38^, ^39^ There may exist some noncoding RNAs or RNA‐binding proteins that bind to the sORF to block the access of ribosome under unstimulated conditions. Under stimulated conditions, these blocking factors are removed, and therefore ribosomes can access to sORF and initiate its translation. The binding and translation of the main ORF may be mediated through a similar mechanism as internal ribosomal entry site.^40^ We cannot exclude the “shift translation” mechanism, which will shift the translation of the 7A‐peptide to the full length HDAC7 protein under some specific circumstances. The overlapped 7A/HDAC7 signal reflects the further splicing of *Hdac7* mRNA to join the sORF and main ORF to produce a full length HDAC7 protein, the translation of which is still initiated from the ATG codon within the sORF. VEGF treatment increased both non‐overlapped and overlapped signals in VPCs, suggesting that VEGF can increase *Hdac7* mRNA further splicing and translation initiation from the ATG codon within the sORF. Further detailed investigation will be required to explore the exact mechanisms involved in the separate translation of the sORF and main ORF. Interestingly, similar sORFs also exist in the predicted human (MHSPGAGWK in *XM_011538479*) and *Heterocephalus glaber* (MHSPGADGTG, XM_004863129) *HDAC7* mRNAs. There are also other sORFs in the 5′‐UTR of all transcript variants. Detailed investigation will be required to explore whether such sORFs are also translatable and what are the underlying mechanisms for the translational regulation of the sORF.

HDAC7 negatively regulates cell migration and proliferation via forming a complex with β‐catenin/E‐cad.^41^, ^42^, ^43^ Mitogenic factors downregulate HDAC7 via transcriptional suppression or posttranslational degradation by protein kinase D‐mediated phosphorylation^35^, ^36^; this process leads to the disruption of HDAC7/β‐catenin/E‐cad. During this process, the kinase undergoes a phosphorylation‐to‐dephosphorylation shift. Phosphorylation usually occurs at serine, threonine, tyrosine, and histidine residues in eukaryotic proteins.^44^, ^45^, ^46^, ^47^ Normally, a kinase has multiple phosphorylation sites, and each site may correspond to a set of substrates. Therefore, a kinase can participate in different cellular processes via receiving phosphate groups at different sites and transferring the phosphate groups to different substrates.

MEKK1 is a serine/threonine kinase that participates in multiple cellular processes, particularly in cell survival and apoptosis regulation.^48^ There are currently two commercially available phosphor‐antibodies for detecting the phosphorylation of MEKK1 at threonine residues 1383 and 1400. In the present study, we identified a new phosphorylation site, serine 393, which was activated by VEGF stimulation. MEKK1 can phosphorylate kinases, such as MKK4,^26^ Raf/ErK,^49^ and JNK^50^, and non‐kinase proteins, such as AXIN1^51^ and 14‐3‐3 proteins.^48^ The 7Aa‐induced MEKK1S393 phosphorylation retention, in which the phosphorylatable serine residue in the 7A peptide is substituted with an unphosphorylatable alanine, suggests that the 7A peptide or 7A‐containing peptides or proteins may be the substrates of MEKK1S393 phosphorylation. During the phosphate group transfer, 7A or 7Aa binds to the MEKK1S393 motif. The adjacent alkaline amino acid may facilitate the access of the serine residue in 7A to the phosphorylated S393 in MEKK1 through an electronic interaction between an NH^+^ and PO_4_^−^. The bond between the hydroxyl group (OH) and PO_4_^−^ in S393 is broken, whereas the bond between OH and PO_4_^−^ in the serine residue in 7A is formed; during this process, the pyrrolidine ring in proline may facilitate the OH/PO_4_^−^ bond disruption and re‐formation, thus transferring the phosphate group from MEKK1S393 to the serine residue in 7A. Phosphorylated 7A undergoes a conformational change, losing its affinity to MEKK1, and thus dissociates from MEKK1. The alanine in 7Aa cannot receive the phosphate group, leaving it stuck to MEKK1, and the PO_4_^−^ remains at the MEKK1S393 site, which is reflected by the increased binding between 7Aa and phosphorylated MEKK1 and the high level of MEKK1S393 phosphorylation. In addition to MEKK1, other kinases may also contribute to 7A phosphorylation, as *Mekk1* knockdown only attenuated but did not totally block 7A phosphorylation. Proteomic analysis also detected other kinases that were associated with the 7A peptide. Detailed investigation will be required.

As described above, MEKK1 can associate with 14‐3‐3 proteins.^48^ Our present study also showed that 14‐3‐3γ phosphorylation at threonine 145 was MEKK1‐dependent because *Mekk1* knockdown abolished VEGF‐induced 14‐3‐3γ phosphorylation at this site. Even more interesting, we found that synthetic phosphor‐7A (7Ap) could facilitate 14‐3‐3γT145 phosphorylation in a cell‐free SDS‐PAGE running gel system. Thus, MEKK1 can phosphorylate 14‐3‐3γ in either a physical interaction‐dependent or interaction‐independent way. In the independent mode, 7Ap acts as the messenger. The 7Ap dependent 14‐3‐3γT145 phosphorylation is also sequence‐dependent, and the adjacent histidine and proline are essential. As the arginine or lysine replacement of histidine does not stop 7A phosphorylation, the serine acts as the receptor residue for the phosphate group. There must be an intramolecular exchange of the phosphate group from the serine residue to the histidine residue because phosphorylated histidine could be detected in synthetic serine‐phosphorylated 7Ap. This intramolecular exchange is essential for the phosphate group transfer from 7Ap to target proteins. Upon binding to the substrate, the histidine residue in 7A may access the hydroxyl group in the threonine residue via NH^+^/OH interaction; during this process, the pyrrolidine ring may facilitate the intramolecular phosphate shift from the serine residue to the N1 site of the imidazole ring of histidine, and the phosphate is then transferred to the OH group within the threonine residue of the target protein. The pyrrolidine ring plays a critical role in the receipt and transfer of the phosphate group, as substitution with valine (which has the same number of carbons) abolishes this process. This might be the first study showing that a small peptide can transfer phosphorylation. It may be reminiscent of the early stage of evolution, when the small peptides and DNA/RNA oligos dominated the earth and evolved into large molecules. Although VEGF activated the MEKK1‐7A‐14‐3‐3γ signal pathway, we also noticed that the exogenous synthetic 7Ap‐mediated 14‐3‐3γ phosphorylation and VPC migration was attenuated by VEGF. This phenomenon provided support on the notion that 14‐3‐3γ phosphorylation contributes to VPC migration on one hand. On the other hand, VEGF may also activate some negative feedback mechanisms to avoid an overreactive MEKK1‐7A‐14‐3‐3γ signal pathway. For example, VEGF may increase the interaction of phosphorylated 7A or 14‐3‐3γ protein with phosphatases like cdc25A.^52^ In *Mekk1si* knockdown VPCs, VEGF had no effect on 7Ap‐mediated migration, different from the control siRNA‐transfected cells. These results suggest that MEKK1 may be also involved in cell migration in 14‐3‐3γ‐independent pathway. Detailed investigation will be required.

Importantly, the endogenous 7A peptide was undetectable in the uninjured femoral arterial wall but was significantly increased in Sca1^+^‐VPCs in response to injury, suggesting that 7A may be translated in only activated progenitor cells. It has been reported that vascular resident stem/progenitor cells contribute to vascular remodeling.^53^, ^54^, ^55^, ^56^ Sca1^+^‐VPCs can differentiate into both ECs and smooth muscle cells.^57^, ^58^ When differentiating toward the EC lineage, Sca1^+^‐VPCs may contribute to vascular injury repair and angiogenesis. Indeed, in this study, we noted that the 7A‐deficient mice had fewer Sca1^+^‐VPCs in the injured vessel wall and ischemic tissues, which led to slower re‐endothelialization in the injured vessels and angiogenesis deterioration in the ischemic tissues. However, local delivery of the exogenous 7A peptide significantly increased the number of Sca1^+^‐VPCs. Therefore, re‐endothelialization and angiogenesis were significantly improved. Thus, this study provided strong evidence for the contribution of Sca1^+^‐VPCs to vascular injury repair and angiogenesis in ischemic tissues. Using different cell surface markers, different groups have identified different types of vessel wall‐resident stem/progenitor cells that contribute to neo‐angiogenesis.^59^, ^60^, ^61^ In this study, we have demonstrated that 7A was essential for neo‐angiogenesis in response to vascular injury. We also detected 7A expression in Sca1^−^ cells. It will be interested to investigate whether 7A is also expressed in the Procr^+^, PW1^+^, or CD157^+^ progenitor cells and involved in the progenitor cells‐mediated neo‐angiogenesis. It will be also worthy to investigate the differentiation of these different types of progenitor cells in vivo via lineage tracing. Denudation of the endothelium is the initial step of atherosclerosis.^62^ Although endothelium denudation was maintained much longer in the injured vessels of the KO mice than in those of the WT mice, we did not observe obvious neointima formation within 3 weeks postsurgery. This finding may be due to the short time period or the decreased number of Sca1^+^‐VPCs in the vessel wall, which may support the notion that local resident progenitor cells contribute to vascular disease development.^55^ Local delivery of the exogenous 7A peptide slightly decreased the media thickness, which may suggest that 7A can suppress smooth muscle cell proliferation. However, a detailed investigation will be required.

Different from the HDAC7 protein, which is indispensable for embryonic development,^9^ the 7A peptide is dispensable, although 7A‐positive cells can be detected at the embryonic stages. 7A‐deficient mice can survive with apparent phenotype of body weight gain under normal diet. As the 7 seconds‐FLAG replacement may affect *Hdac7* mRNA transcript variant 2 undergoing further splicing, we cannot exclude the possibility that some of the phenotypes observed may be due to the lack of full‐length HDAC7 protein. Some of the phenotypes may be due to lack of 7A, some due to lack of full‐length HDAC7, and some due to the combined effect from the lack of both 7A and full‐length HDAC7 protein. The transgenic mice will also serve a good model to investigate the function of full‐length HDAC7 protein, especially in heart functions, as we have detected the full length of HDAC7 in coronary arteries in wild‐type mice. However, the rescue effect by the exogenous 7A‐peptide still provides strong evidence for the endogenous 7A‐mediated functions. Considering that 7A was detected in only the injured vessel walls and that 7A deficiency considerably slowed injured vessel re‐endothelialization and reduced angiogenesis in ischemic tissues, we can assume that the alternative translation of the 7A peptide is a pathological response. Therefore, the exogenous 7A peptide and its more active form, 7Ap, may possess therapeutic potential for vascular injury or angiogenesis‐related disease intervention.

In summary, VEGF or pathological stimulus induces translation of the sORF from *Hdac7* mRNA in Sca1^+^‐VPCs. On the one hand, this process produces a 7‐aa peptide (7A). On the other hand, the exogenous stimulus activates MEKK1 via phosphorylation at the Ser393 residue. Activated MEKK1 transfers the phosphate group from Ser393 to the serine residue of the 7‐aa peptide. The intramolecular serine‐to‐histidine phosphate transfer may affect 7Ap confirmation and therefore affect its affinity to 14‐3‐3γ protein, facilitating its phosphorylation, leading to the cadherin‐plakoglobin‐catenin‐14‐3‐3γ complex disruption and 14‐3‐3γ nuclear translocation. Other signaling pathways may also contribute to and/or be activated by 7A phosphorylation. The overall effect leads to Sca1^+^‐VPC migration and differentiation toward the EC lineage; these effects contribute to vascular injury repair and angiogenesis in ischemic tissue (Figure S10). The significance of this study is that small peptides, such as 7A, can play an important role in multiple physiological and pathological processes via functioning as novel signaling messengers to expand the substrate spectrum of kinases.

## CONFLICT OF INTERESTS

The authors declare no potential conflict of interest.

## AUTHOR CONTRIBUTIONS

J.Y., A. Moraga: contributed to experimental design, performance, data analysis, paper writing; J.X., Y.Z, P.L, W.D., K.H.L., A. Margariti, M.Z., Z.Z., Y.H.: contributed to experimental performance; Q.Z., G.W., L. Zheng: contributed to data analysis; W.W., L.S., A.S., A.M.S., Q.W.: contributed to manuscript writing; L. Zeng: contributed to experimental design, data analysis, manuscript writing. All authors were involved in critical evaluation and intellectual contribution to the manuscript.

## Supporting information


**Appendix** **S1.** Supporting Information.

## Data Availability

The data that support the findings of this study are available on request from the corresponding author. The data are not publicly available due to privacy or ethical restrictions.

## References

[1] Avorn J , Knight E , Ganz DA , Schneeweiss S . Therapeutic delay and reduced functional status six months after thrombolysis for acute myocardial infarction. Am J Cardiol. 2004;94:415‐420. 10.1016/j.amjcard.2004.04.055. 15325921

[2] Park KH , Park WJ . Endothelial dysfunction: clinical implications in cardiovascular disease and therapeutic approaches. J Korean Med Sci. 2015;30:1213‐1225. 10.3346/jkms.2015.30.9.1213. 26339159 PMC4553666

[3] Libby P . Inflammation in atherosclerosis. Arterioscler Thromb Vasc Biol. 2012;32:2045‐2051. 10.1161/ATVBAHA.108.179705. 22895665 PMC3422754

[4] Xu Q , Zhang Z , Davison F , Hu Y . Circulating progenitor cells regenerate endothelium of vein graft atherosclerosis, which is diminished in ApoE‐deficient mice. Circ Res. 2003;93:e76‐e86. 10.1161/01.RES.0000097864.24725.60. 14512446

[5] Fanger GR et al. 14–3‐3 proteins interact with specific MEK kinases. J Biol Chem. 1998;273:3476‐3483. 10.1074/jbc.273.6.3476. 9452471

[6] Zengin E et al. Vascular wall resident progenitor cells: a source for postnatal vasculogenesis. Development. 2006;133:1543‐1551. 10.1242/dev.02315. 16524930

[7] Kornblihtt AR et al. Alternative splicing: a pivotal step between eukaryotic transcription and translation. Nat Rev Mol Cell Biol. 2013;14:153‐165. 10.1038/nrm3525. 23385723

[8] Lee S et al. Global mapping of translation initiation sites in mammalian cells at single‐nucleotide resolution. Proc Natl Acad Sci U S A. 2012;109:E2424‐E2432. 10.1073/pnas.1207846109. 22927429 PMC3443142

[9] Chang S et al. Histone deacetylase 7 maintains vascular integrity by repressing matrix metalloproteinase 10. Cell. 2006;126:321‐334. 10.1016/j.cell.2006.05.040. 16873063

[10] Fischle W et al. A new family of human histone deacetylases related to *Saccharomyces cerevisiae* HDA1p. J Biol Chem. 1999;274:11713‐11720. 10.1074/jbc.274.17.11713. 10206986

[11] Ummarino D , Zeng L . Is C reactive protein expression affected by local microenvironment? Heart. 2013;99:514‐515. 10.1136/heartjnl-2012-303436. 23343683

[12] Ni Z et al. Recipient c‐kit lineage cells repopulate smooth muscle cells of transplant arteriosclerosis in mouse models. Circ Res. 2019;125:223‐241. 10.1161/CIRCRESAHA.119.314855. 31079549 PMC6615935

[13] Xie Y et al. Leptin induces Sca‐1(+) progenitor cell migration enhancing Neointimal lesions in vessel‐injury mouse models. Arterioscler Thromb Vasc Biol. 2017;37:2114‐2127. 10.1161/ATVBAHA.117.309852. 28935755 PMC5671780

[14] Le Bras A et al. Adventitial Sca1+ cells transduced with ETV2 are committed to the endothelial fate and improve vascular Remodeling after injury. Arterioscler Thromb Vasc Biol. 2018;38:232‐244. 10.1161/ATVBAHA.117.309853. 29191922 PMC5757665

[15] Zeng L et al. XBP 1‐deficiency abrogates neointimal lesion of injured vessels via cross talk with the PDGF Signaling. Arterioscler Thromb Vasc Biol. 2015;35:2134‐2144. 10.1161/ATVBAHA.115.305420. 26315405

[16] Zeng L et al. Vascular endothelial cell growth‐activated XBP1 splicing in endothelial cells is crucial for angiogenesis. Circulation. 2013;127:1712‐1722. 10.1161/CIRCULATIONAHA.112.001337. 23529610

[17] Margariti A et al. Splicing of HDAC7 modulates the SRF‐myocardin complex during stem‐cell differentiation towards smooth muscle cells. J Cell Sci. 2009;122:460‐470. 10.1242/jcs.034850. 19174469

[18] Tsai TN et al. Contribution of stem cells to neointimal formation of decellularized vessel grafts in a novel mouse model. Am J Pathol. 2012;181:362‐373. 10.1016/j.ajpath.2012.03.021. 22613026

[19] Sendoel A et al. Translation from unconventional 5′ start sites drives tumour initiation. Nature. 2017;541:494‐499. 10.1038/nature21036. 28077873 PMC5287289

[20] Aird WC . Endothelial cell heterogeneity. Cold Spring Harb Perspect Med. 2012;2:a006429 10.1101/cshperspect.a006429. 22315715 PMC3253027

[21] Pan Y et al. Histone Deacetylase 7‐derived peptides play a vital role in vascular repair and regeneration. Adv Sci (Weinh). 2018;5:1800006 10.1002/advs.201800006. 30128229 PMC6097091

[22] Zhang Y et al. A collagen hydrogel loaded with HDAC7‐derived peptide promotes the regeneration of infarcted myocardium with functional improvement in a rodent model. Acta Biomater. 2019;86:223‐234. 10.1016/j.actbio.2019.01.022. 30660010

[23] Margariti A et al. Histone deacetylase 7 controls endothelial cell growth through modulation of beta‐catenin. Circ Res. 2010;106:1202‐1211. 10.1161/CIRCRESAHA.109.213165. 20224040

[24] Klumpp S , Krieglstein J . Reversible phosphorylation of histidine residues in vertebrate proteins. Biochim Biophys Acta. 2005;1754:291‐295. 10.1016/j.bbapap.2005.07.035. 16194631

[25] Muimo R et al. Histidine phosphorylation of annexin I in airway epithelia. J Biol Chem. 2000;275:36632‐36636. 10.1074/jbc.M000829200. 10956639

[26] Owen GR , Achilonu I , Dirr HW . High yield purification of JNK1beta1 and activation by in vitro reconstitution of the MEKK1–>MKK4–>JNK MAPK phosphorylation cascade. Protein Expr Purif. 2013;87:87‐99. 10.1016/j.pep.2012.10.010. 23147205

[27] Yu B et al. Vascular stem/progenitor cell migration induced by smooth muscle cell‐derived chemokine (C‐C motif) ligand 2 and chemokine (C‐X‐C motif) ligand 1 contributes to Neointima formation. Stem Cells. 2016;34:2368‐2380. 10.1002/stem.2410. 27300479 PMC5026058

[28] Boulant S , Becchi M , Penin F , Lavergne JP . Unusual multiple recoding events leading to alternative forms of hepatitis C virus core protein from genotype 1b. J Biol Chem. 2003;278:45785‐45792. 10.1074/jbc.M307174200. 12952944

[29] Chu Q , Ma J , Saghatelian A . Identification and characterization of sORF‐encoded polypeptides. Crit Rev Biochem Mol Biol. 2015;50:134‐141. 10.3109/10409238.2015.1016215. 25857697 PMC4761265

[30] Cleary JD , Ranum LP . Repeat associated non‐ATG (RAN) translation: new starts in microsatellite expansion disorders. Curr Opin Genet Dev. 2014;26:6‐15. 10.1016/j.gde.2014.03.002. 24852074 PMC4237677

[31] Powell ML , Brown TD , Brierley I . Translational termination‐re‐initiation in viral systems. Biochem Soc Trans. 2008;36:717‐722. 10.1042/BST0360717. 18631147

[32] Andrews SJ , Rothnagel JA . Emerging evidence for functional peptides encoded by short open reading frames. Nat Rev Genet. 2014;15:193‐204. 10.1038/nrg3520. 24514441

[33] Plaza S , Menschaert G , Payre F . In search of lost small peptides. Annu Rev Cell Dev Biol. 2017;33:391‐416. 10.1146/annurev-cellbio-100616-060516. 28759257

[34] Anderson DM et al. A micropeptide encoded by a putative long noncoding RNA regulates muscle performance. Cell. 2015;160:595‐606. 10.1016/j.cell.2015.01.009. 25640239 PMC4356254

[35] Sinnett‐Smith J et al. Protein kinase D1 mediates class IIa histone deacetylase phosphorylation and nuclear extrusion in intestinal epithelial cells: role in mitogenic signaling. Am J Physiol Cell Physiol. 2014;306:C961‐C971. 10.1152/ajpcell.00048.2014. 24647541 PMC4024715

[36] Wang S et al. Control of endothelial cell proliferation and migration by VEGF signaling to histone deacetylase 7. Proc Natl Acad Sci U S A. 2008;105:7738‐7743. 10.1073/pnas.0802857105. 18509061 PMC2409381

[37] Fabrega C , Hausmann S , Shen V , Shuman S , Lima CD . Structure and mechanism of mRNA cap (guanine‐N7) methyltransferase. Mol Cell. 2004;13:77‐89. 14731396 10.1016/s1097-2765(03)00522-7

[38] Molina‐Garcia L , Giraldo R . Enabling stop codon read‐through translation in bacteria as a probe for amyloid aggregation. Sci Rep. 2017;7:11908 10.1038/s41598-017-12174-0. 28928456 PMC5605706

[39] Powell ML , Napthine S , Jackson RJ , Brierley I , Brown TD . Characterization of the termination‐reinitiation strategy employed in the expression of influenza B virus BM2 protein. RNA. 2008;14:2394‐2406. 10.1261/rna.1231008. 18824510 PMC2578862

[40] Hashem Y et al. Hepatitis‐C‐virus‐like internal ribosome entry sites displace eIF3 to gain access to the 40S subunit. Nature. 2013;503:539‐543. 10.1038/nature12658. 24185006 PMC4106463

[41] Bradley EW , Carpio LR , Olson EN , Westendorf JJ . Histone deacetylase 7 (Hdac7) suppresses chondrocyte proliferation and beta‐catenin activity during endochondral ossification. J Biol Chem. 2015;290:118‐126. 10.1074/jbc.M114.596247. 25389289 PMC4281714

[42] Chavdoula ED , Panagopoulos DJ , Margaritis LH . Comparison of biological effects between continuous and intermittent exposure to GSM‐900‐MHz mobile phone radiation: detection of apoptotic cell‐death features. Mutat Res. 2010;700:51‐61. 10.1016/j.mrgentox.2010.05.008. 20472095

[43] Zhou B et al. Splicing of histone deacetylase 7 modulates smooth muscle cell proliferation and neointima formation through nuclear beta‐catenin translocation. Arterioscler Thromb Vasc Biol. 2011;31:2676‐2684. 10.1161/ATVBAHA.111.230888. 21836063

[44] Burnett G , Kennedy EP . The enzymatic phosphorylation of proteins. J Biol Chem. 1954;211:969‐980. 13221602

[45] Chang C , Stewart RC . The two‐component system. Regulation of diverse signaling pathways in prokaryotes and eukaryotes. Plant Physiol. 1998;117:723‐731. 10.1104/pp.117.3.723. 9662515 PMC1539182

[46] Cozzone AJ . Protein phosphorylation in prokaryotes. Annu Rev Microbiol. 1988;42:97‐125. 10.1146/annurev.mi.42.100188.000525. 2849375

[47] Stock JB , Ninfa AJ , Stock AM . Protein phosphorylation and regulation of adaptive responses in bacteria. Microbiol Rev. 1989;53:450‐490. 2556636 10.1128/mr.53.4.450-490.1989 PMC372749

[48] Schlesinger TK , Fanger GR , Yujiri T , Johnson GL . The TAO of MEKK. Front Biosci. 1998;3:D1181‐D1186. 9820741 10.2741/a354

[49] Karandikar M , Xu S , Cobb MH . MEKK1 binds raf‐1 and the ERK2 cascade components. J Biol Chem. 2000;275:40120‐40127. 10.1074/jbc.M005926200. 10969079

[50] Baud V et al. Signaling by proinflammatory cytokines: oligomerization of TRAF2 and TRAF6 is sufficient for JNK and IKK activation and target gene induction via an amino‐terminal effector domain. Genes Dev. 1999;13:1297‐1308. 10.1101/gad.13.10.1297. 10346818 PMC316725

[51] Zhang Y et al. Casein kinase I and casein kinase II differentially regulate axin function in Wnt and JNK pathways. J Biol Chem. 2002;277:17706‐17712. 10.1074/jbc.M111982200. 11884395

[52] Kasahara K et al. 14–3‐3gamma mediates Cdc25A proteolysis to block premature mitotic entry after DNA damage. EMBO J. 2010;29:2802‐2812. 10.1038/emboj.2010.157. 20639859 PMC2924644

[53] Hu Y et al. Abundant progenitor cells in the adventitia contribute to atherosclerosis of vein grafts in ApoE‐deficient mice. J Clin Investig. 2004;113:1258‐1265. 10.1172/JCI19628. 15124016 PMC398426

[54] Majesky MW , Dong XR , Hoglund V , Mahoney WM Jr , Daum G . The adventitia: a dynamic interface containing resident progenitor cells. Arterioscler Thromb Vasc Biol. 2011;31:1530‐1539. 10.1161/ATVBAHA.110.221549. 21677296 PMC3382115

[55] Tang Z et al. Differentiation of multipotent vascular stem cells contributes to vascular diseases. Nat Commun. 2012;3:875 10.1038/ncomms1867. 22673902 PMC3538044

[56] Torsney E , Xu Q . Resident vascular progenitor cells. J Mol Cell Cardiol. 2011;50:304‐311. 10.1016/j.yjmcc.2010.09.006. 20850452

[57] Xiao Q , Zeng L , Zhang Z , Hu Y , Xu Q . Stem cell‐derived Sca‐1+ progenitors differentiate into smooth muscle cells, which is mediated by collagen IV‐integrin alpha1/beta1/alphav and PDGF receptor pathways. Am J Physiol Cell Physiol. 2007;292:C342‐C352. 10.1152/ajpcell.00341.2006. 16914533

[58] Xiao Q et al. Sca‐1+ progenitors derived from embryonic stem cells differentiate into endothelial cells capable of vascular repair after arterial injury. Arterioscler Thromb Vasc Biol. 2006;26:2244‐2251. 10.1161/01.ATV.0000240251.50215.50. 16902164

[59] Malinverno M et al. Peg3/PW1 is a marker of a subset of vessel associated endothelial progenitors. Stem Cells. 2017;35:1328‐1340. 10.1002/stem.2566. 28090691

[60] Wakabayashi T et al. CD157 marks tissue‐resident endothelial stem cells with homeostatic and regenerative properties. Cell Stem Cell. 2018;22:384‐397 e386. 10.1016/j.stem.2018.01.010. 29429943

[61] Yu QC , Song W , Wang D , Zeng YA . Identification of blood vascular endothelial stem cells by the expression of protein C receptor. Cell Res. 2016;26:1079‐1098. 10.1038/cr.2016.85. 27364685 PMC5113308

[62] Ross R . Atherosclerosis–an inflammatory disease. N Engl J Med. 1999;340:115‐126. 10.1056/NEJM199901143400207. 9887164

