# Supplementary material for: A histone deacetylase 7‐derived peptide promotes vascular regeneration via facilitating 14‐3‐3γ phosphorylation
Source: Stem Cells. 2020 Jan 29;38(4):556–73. doi: 10.1002/stem.3122 (PMC7187271; doi:10.1002/stem.3122)
Supplement: Supplementary file 1 — Appendix S1. Supporting Information. [file STEM-38-556-s001.pdf]

Supplemental Figure Legends

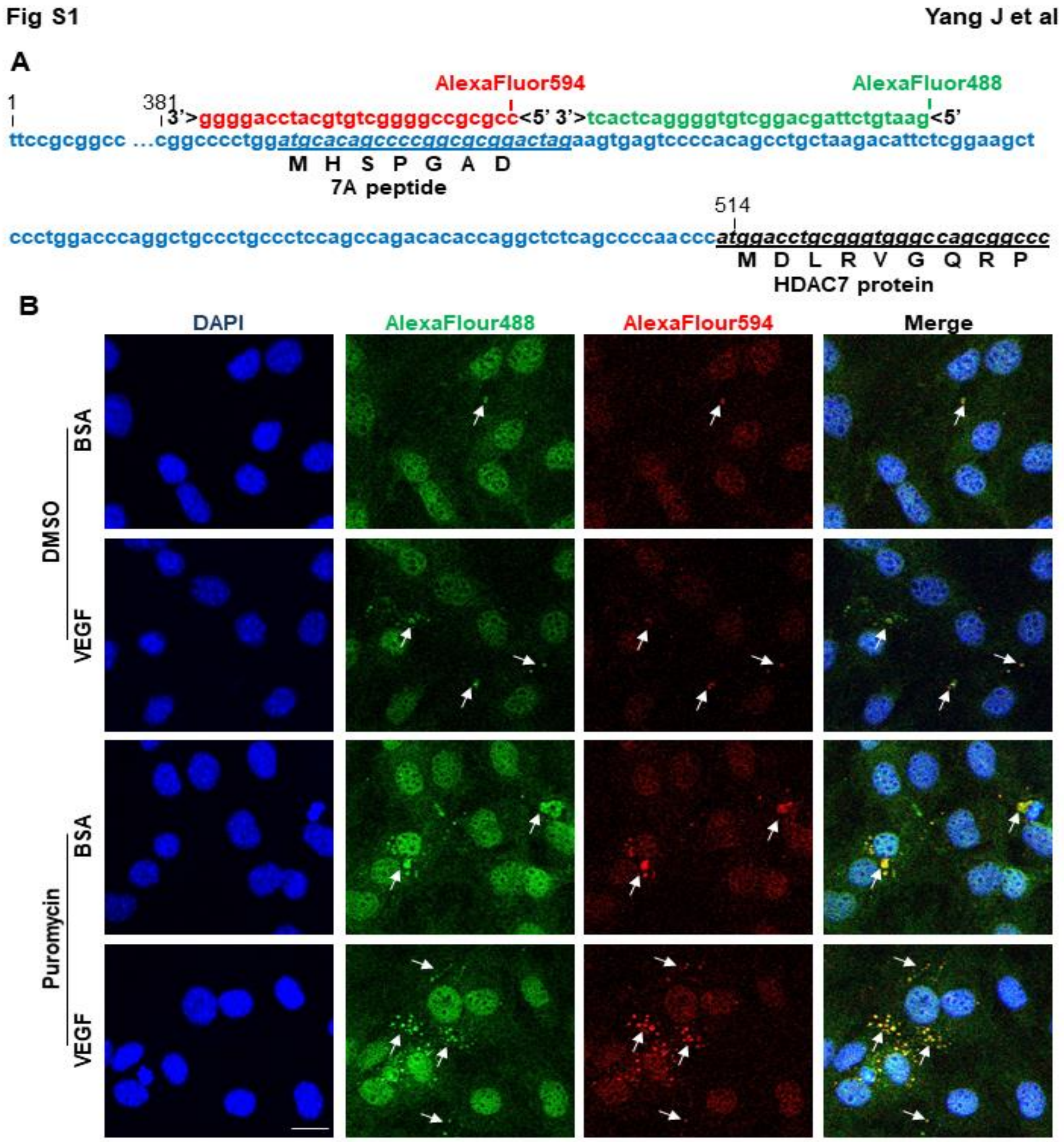

Figure S1: The translation of the short open reading frame was demonstrated by FISH experiments. (A) A schematic illustration of the 5'terminal untranslated region (in blue) of mouse Hdac7 mRNA (NM_001204276.1) *and the short open reading frame*. The short and main open reading frames were italic in bold with amino acid sequences listed below the sequences and marked as 7A peptide and HDAC7 protein, respectively. The Alexa Fluor 488 (green) and 594 (red)-labelled DNA oligo sequences were placed upon the complementary sequences, respectively. Number indicates the nucleotide position. **(B)** Sca1^{+}-VPCs (2x10^{5}) were treated with 5ng/mL VEGF in serum free medium for 30 min with 10μg/mL puromycin included in the last 15 min, followed by hybridization with AlexFluor488 and AlexaFluor594-labelled DNA probes as illustrated above. Arrows show the overlapped signals. Scale bar: 5μm. Data presented were representative images of three independent experiments.

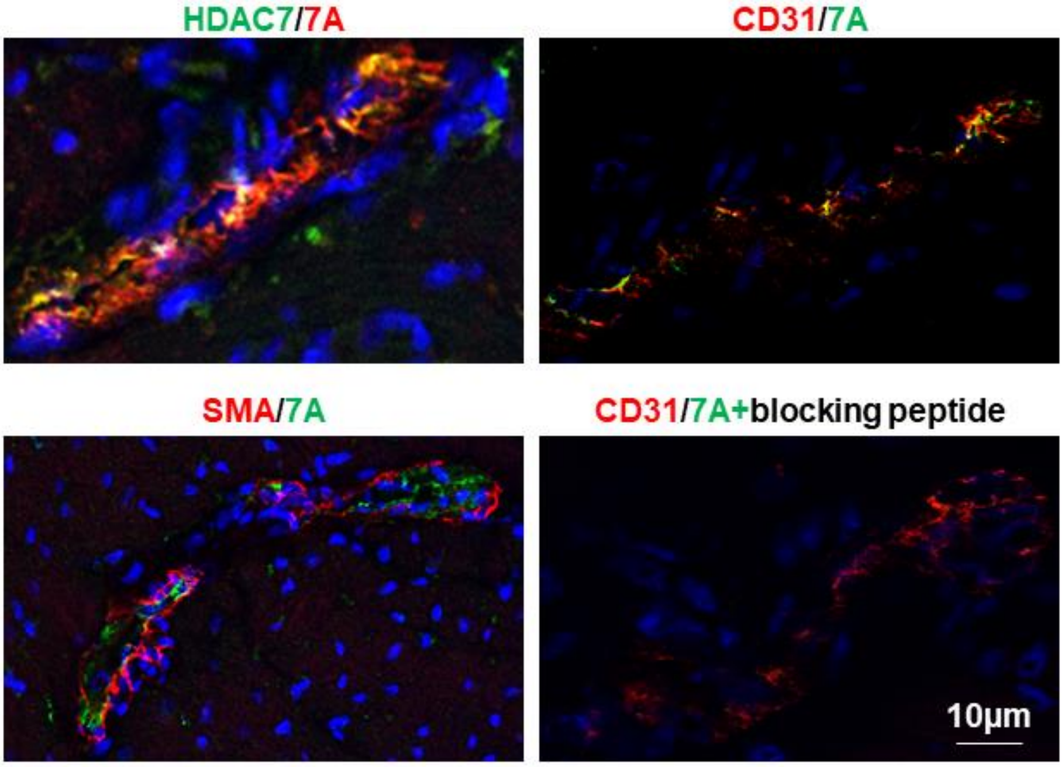

Figure S2: 7A was expressed in endothelium in the heart. Left ventricular tissues were harvested from 8 weeks C57bl mice. Double immunofluorescence staining was performed on cryosections with antibodies indicated. The colours indicate the relative secondary antibodies used. For the blocking assay, anti-7A antibody was pre-incubated with 7A peptide at 1:1 (μg) overnight prior to the experiments. Note that 7A was expressed in endothelial cells and some of the 7A signal was overlapped in HDAC7 signal, indicating 7A exists in the N-terminal of HDAC7 protein. The data presented were representative images from six mice.

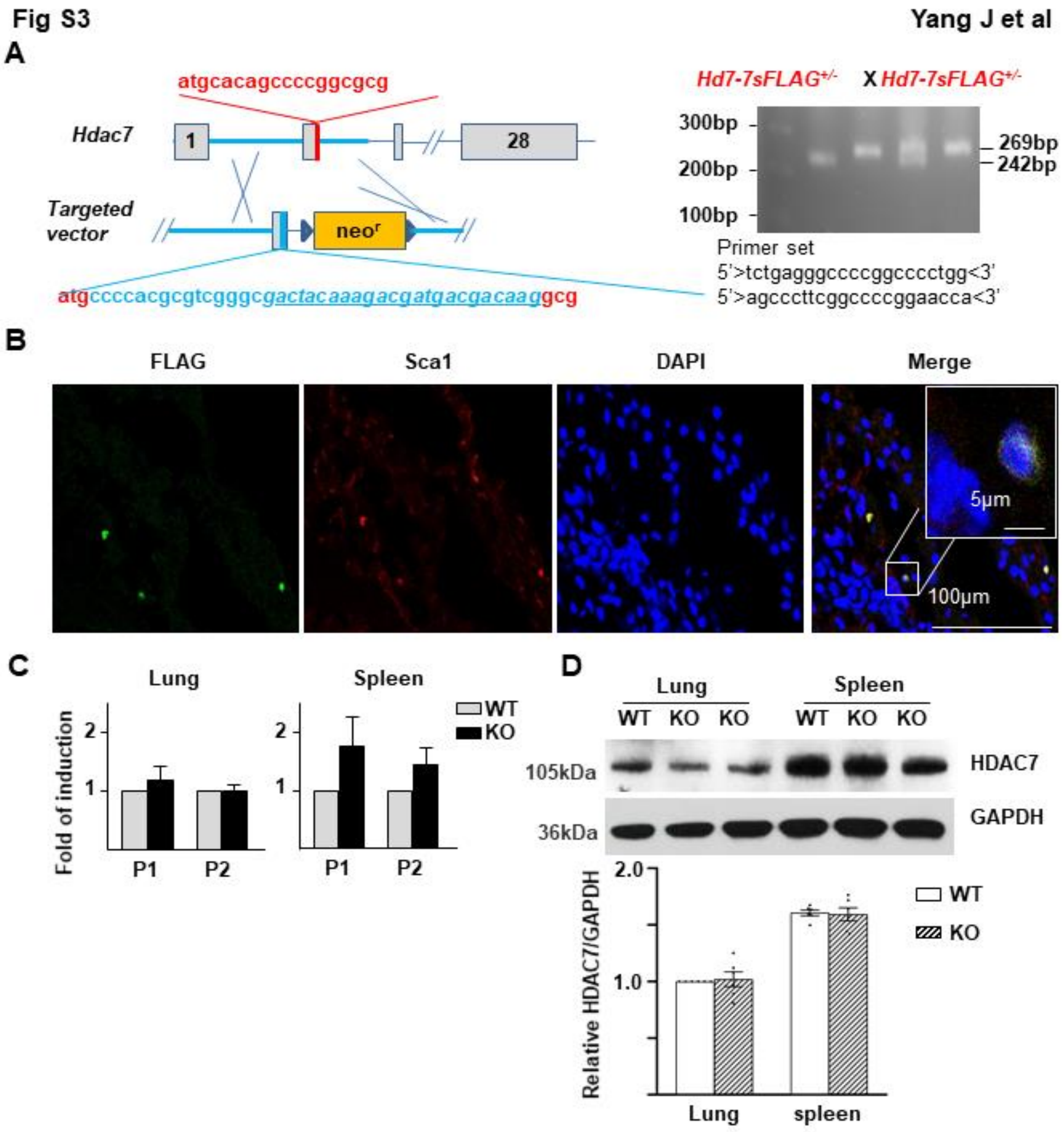

Figure S3: 7A deficiency had no effect on Hdac7 expression. (A) A schematic illustration of the creation of *Hd7-7sFLAG* transgenic mice (Left) and genotyping (Right). The 7A sequence in exon 2 was replaced with 7S sequence that was tagged with a FLAG sequence (in italic and underlined). **(B)** Double immunofluorescence staining of FLAG and Sca1 on cryo-sections of the aorta isolated from *Hd7-7sFLAG*$^{+/-}$ mice. (**C, D**) 7A deficiency had no effect on Hdac7 expression. Cellular total RNA and proteins were isolated from wild type (WT, n=3) and *Hd7-7sFLAG*$^{+/+}$ transgenic (KO, n=6) mice lung and spleen tissues, subjected to quantitative RT-PCR (**C**) or Western blot (**D**). The primer sets *5'>ctg aga gcc tgg tgt gtc tgg ctg<3'* vs 5'>*tgt ctc ctt tct cag gct gct ctc*<3' and *5'>ctg aga gcc tgg tgt gtc tgg ctg<3'* vs 5'>*tct cgc cgt ctc aca gtc gct ctg*<3' were used to amplify *Hdac7* mRNA transcript variants transcribed from promoter 1 (P1) and 2 (P2), respectively. The primer set for β-actin (5'>*cac aac tgg gac gac atg gag<3' and 5'>ttc aga ggt agt cag tct gg<3'*) was included as internal control. The fold of induction was defined as the ratio of KO to WT with that of WT set as 1.0. Data presented were representative images or mean of three independent experiments.

Fig S4 Yang J et al

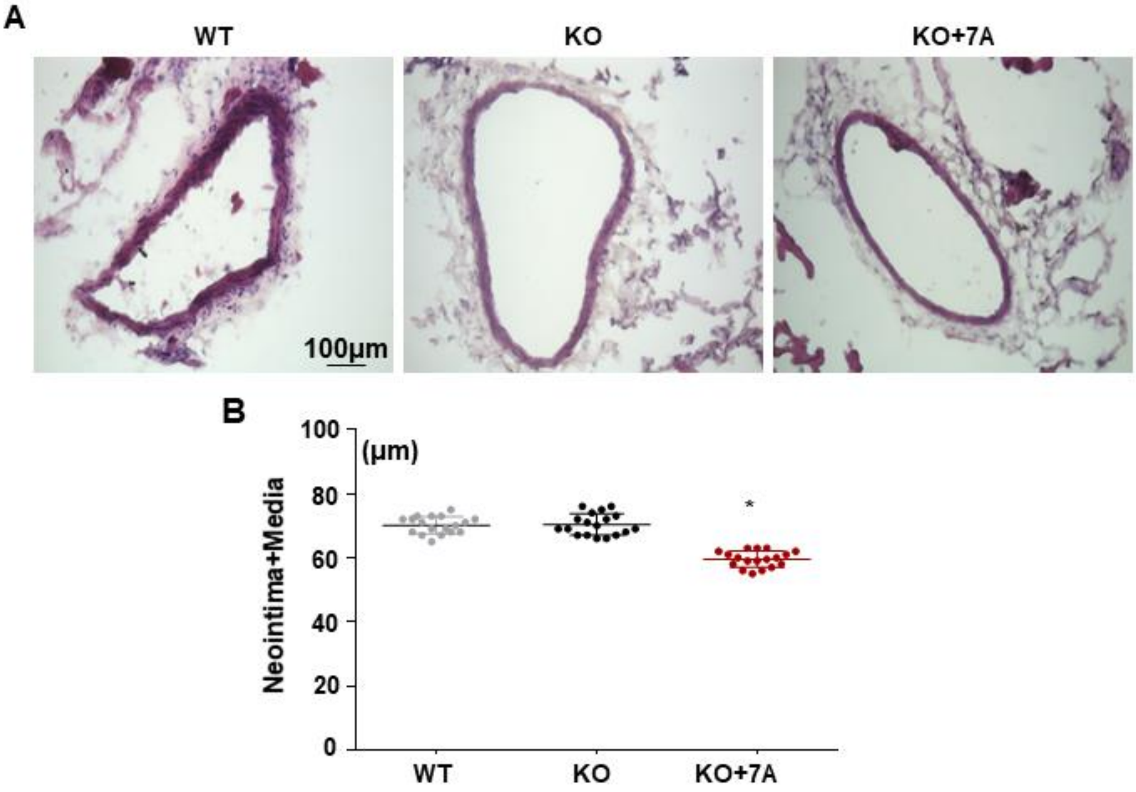

Figure S4: 7A reduced vessel wall thickness in 7-aa peptide deficient mice. Femoral artery injury model was introduced wild type C57Bl/6 mice (WT) and *Hd7-7sFLAG*$^{+/+}$ mice (KO) via platinum wire scratching. For half of the KO mice, 200μl of Pluronic F-127 gel containing 10ng/ml 7A peptide was applied around the injured vessel (KO+7A). Three weeks post surgery, the injured vessels were harvested and cryo-sectioned, followed by H&E staining (**A**). The average intima+media thickness was calculated with sections from the central part of the isolated vessels. For each section, 6 points were calculated. Data are presented as mean ± SD, n =18 section per experimental group from 6 mice. Statistics was done using two-way ANOVA test followed by Tukey's multiple comparisons test. *: $p<0.05$.

Fig S5 **Yang J et al**

A

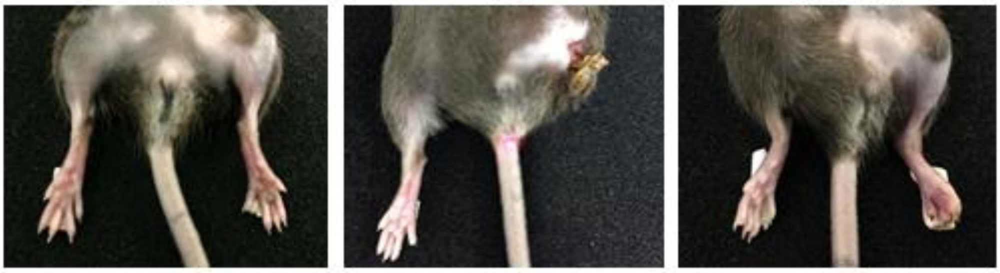

B

	Day 7			Day 14		
	WT	KO	KO+7A	WT	KO	KO+7A
minimal	-	-	-	6/6	-	-
mild	6/6	-	2/6	-	-	4/6
moderate	-	2/6	4/6	-	4/6	2/6
M/S	-	4/6	-	-	-	-
severe	-	-	-	-	2/6	-

- **Minimal:** the foot looks the same than the uninjured one.
- **Mild:** the foot is slightly crooked and the movement is affected.
- **Moderate:** the foot is completely crooked and the foot is dragged
- **Severe:** the foot is lost.

Figure S5: 7A deficiency increased foot necrosis in the hindlimb ischemia model. Hindlimb ischemia model was introduced in wild type (WT) and Hd7-7sFLAG$^{+/+}$ transgenic mice (KO). Local delivery of 7A peptide was applied via 200µl of pluronic gel containing 10ng/ml 7A peptide (KO+7A). Necrosis was observed at day 7 and day 14 post-surgery. (**A**) images show the necrosis scale. (**B**) Quantitative analysis. M/S: moderate to severe.

Fig S6

Yang J et al

A

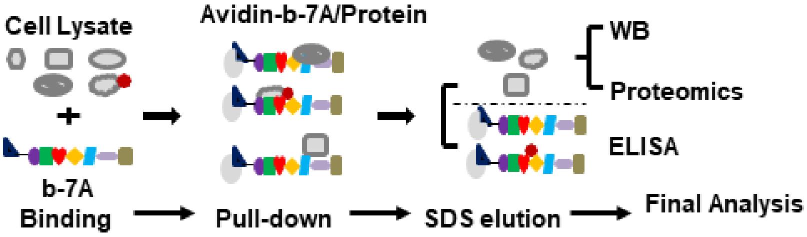

B

Name	Sequence	Name	Sequence
7S	MPHASGD	b-7Ar	Biotin-MRSPGAD
7A	MHSPGAD	b-7Al	Biotin-MLSPGAD
7Aa	MHAPGAD	b-7At	Biotin-MTSPGAD
7Ak	MKSPGAD	b-7Av	Biotin-MHSVGAD
7Ar	MRSPGAD	7Sp	MPHA[pS]GD
7Al	MLSPGAD	7Ap	MH[pS]PGAD
7At	MTSPGAD	7Akp	MK[pS]PGAD
7Av	MHSVGAD	7Arp	MR[pS]PGAD
b-7S	Biotin-MPHASGD	7Alp	ML[pS]PGAD
b-7A	Biotin-MHSPGAD	7Atp	MT[pS]PGAD
b-7Aa	Biotin-MHAPGAD	7Avp	MH[pS]VGAD
b-7Ap	Biotin-MH[pS]PGAD	7Ap-b	MH[pS]GADK-Biotin
b-7Ak	Biotin-MKSPGAD		

Figure S6: A schematic illustration of peptide/protein binding assay and a list the 7aa peptide and derivatives. (A) The biotin labelled 7A (b-7A) was incubated with cell lysate. The b-7A and its associated proteins were pulled down by streptavidin magnetic beads. The non-specific binding proteins were washed away with 0.5% Triton X-100 containing washing buffer. The specific associated proteins were eluted with SDS-containing buffer and subjected to Western blot (WB) or proteomics analysis. The beads containing b-7A were subjected to ELISA with anti-phospho-Serine antibody to detect 7A phosphorylation. **(B)** A list of the 7aa peptides and their sequences. [pS]: phospho-serine.

Fig S7

Yang J et al

A

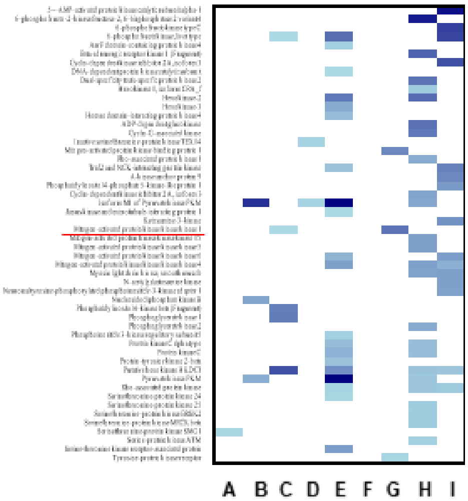

B

		BSA				VEGF			
		b-7S	b-7Aa	b-7A	b-7Ap	b-7S	b-7Aa	b-7A	b-7Ap
MEKK 1	t		2				2		
	p		1				1		
MEKK 3	t							2	
	p								
MEKK 4	t				4			2	4
	p				1				
MEKK 5	t				2				
	p				1				
MEKK 15	t				6				
	p				2				

Figure S7. Kinases identified by proteomics analysis. Sca1^{+}-VPCs were incubated with serum free medium for 4hr, then treated with 5ng/ml VEGF (dissolved in 1% BSA) and 10u/ml heparin for 30min. Fifty µg of cell lysate was incubated with 5µg of biotin-labelled 7-aa peptides (b-7x) at 37ºC for 1hr. Streptavidin magnetic beads was then added and incubated at 4ºC for 1hr. The peptide-associated proteins were eluted with SDS-containing solutions and subjected to proteomics analysis. **(A)** Heatmap and **(B)** the list of identified pospho-kinases by proteomics analysis, t: number of the total peptides detected; p: number of phospho-peptides detected. A: Control, B: b-7s, C: b-7Aa, D: b-7A, E: b-7Ap, F: VEGF treated b-7s, G: VEGF treated b-7Aa, H:VEGF treated b-7A, I: VEGF treated b-7Ap.

Fig S8

Yang J et al

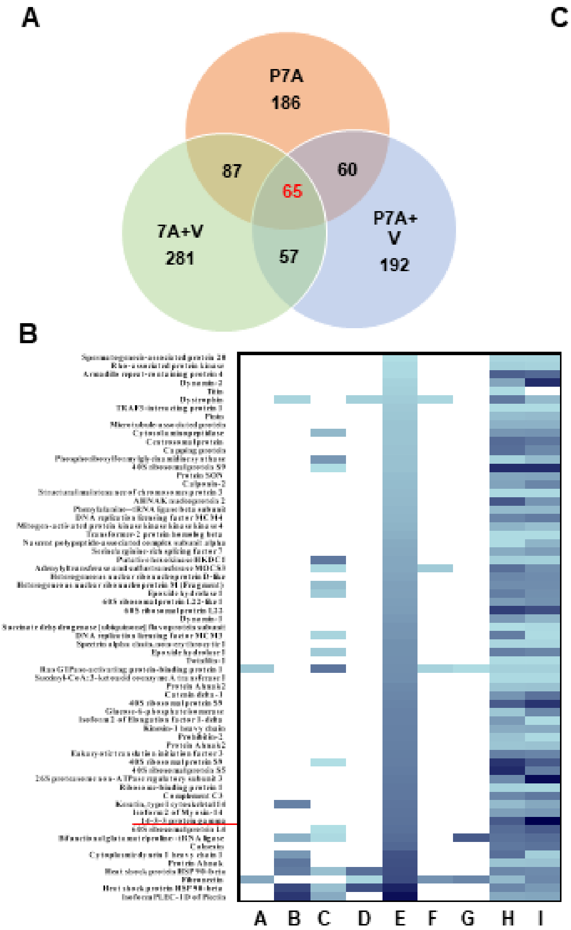

C

		BSA				VEGF			
		b-7S	b-7Aa	b-7A	b-7Ap	b-7S	b-7Aa	b-7A	b-7Ap
CTNNA1	t		6		14			4	4
	p								
CTNNA2	t				5			5	
	p								
CTNNB1	t		5		5		2	12	12
	p		1				1		1
CTNND1	t		2		19			39	18
	p				2			3	
1433 α/β	t	5	3		7			4	6
	p								
1433 γ	t		7		10			8	6
	p				1			1	1
1433 ζ	t	4	5	4	6	2		4	4
	p								
1433 ε	t	3	5		7	1	3	3	4
	p								
1433 σ	t	3	4		4	1		2	3
	p								
1433 tau	t	5	7	1	5	1	1	2	5
	p								

Figure S8. The components of E-cadherin complex identified by proteomics analysis. Sca1^{+}-VPCs were incubated with serum free medium for 4hr, then treated with 5ng/ml VEGF (dissolved in 1% BSA) and 10u/ml heparin for 30min. Fifty µg of cell lysate was incubated with 5µg of biotin-labelled 7-aa peptides (b-7x) at 37ºC for 1hr. Streptavidin-agarose beads was then added and incubated at 4ºC for 1hr. The peptide-associated proteins were eluted with SDS-containing solutions and subjected to proteomics analysis. Venn Diagram of phospho-proteins of 7A+VEGF(green cycle), p7A(orange cycle) and p7A+VEGF(blue cycle) , the 65 overlapped proteins was showed in heatmap (B). The components of E-cadherin complex was

shown on the list (C). t: number of the total peptides detected; p: number of phospho-peptides detected. A: Control, B: b-7s, C: b-7Aa, D: b-7A, E: b-7Ap, F: VEGF treated b-7s, G: VEGF treated b-7Aa, H:VEGF treated b-7A, I: VEGF treated b-7Ap.

Fig S9

Yang J et al

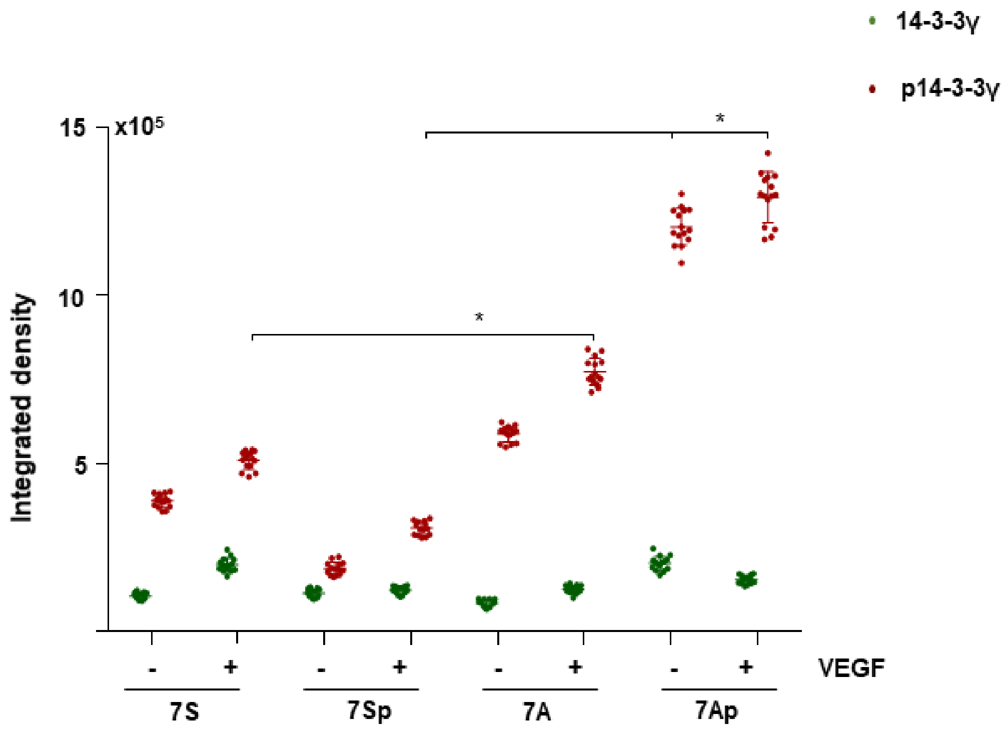

Figure S9: Quantification of the immunofluorescence staining for Figure 4F. The $Sca1^+$-VPCs ($1x10^6$) were pre-treated with 1ng/mL 7S or 7Sp (phosphorylated 7S) or 7A or 7Ap for 1hr, treated with 5ng/mL VEGF in the presence of the peptides for 30min, and subject to immunofluorescence staining with anti-phospho-14-3-3γThr145 (p14-3-3γ) and anti-14-3-3γ antibodies. The fluorescence density was measured from 30 nuclei for each group using ImageJ software. PBS was included as a control for peptides or biotin-labeled peptides. BSA was used as a vehicle control for VEGF treatment. Data are presented as mean ± SD, n =15 cell cultures per experimental group from three individual preparations. Statistics was done using the two-way ANOVA test followed by Tukey's multiple comparisons test. *: $p<0.05$.

Fig. S10 Yang J et al

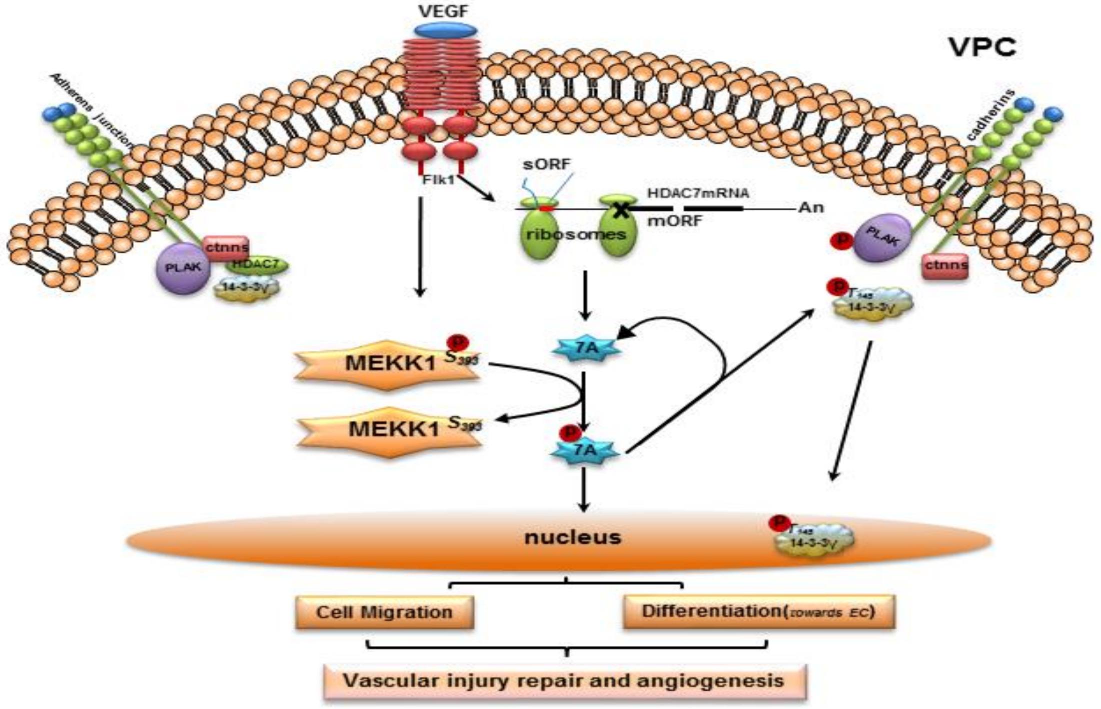

Figure S10: A schematic illustration of VEGF-induced HDAC7 alternative translation and its role in signal transduction. VEGF binds to its receptor Flk1, triggering the translation of the sORF, giving rise to a 7-aa peptide (7A). Flk1 activates MEKK1 phosphorylation at Ser393. The phosphorylated MEKK1 transfers the phosphate group from Ser393 to the serine residue of 7A, forming phosphorylated 7A (7Ap). Then, 7Ap transfers the phosphate group to the Thr145 site within 14-3-3γ, leading to 14-3-3γ nuclear translocation. The overall effect leads to Sca1^{+}-VPC migration and differentiation towards EC lineage, contributing to vascular injury repair and angiogenesis in ischemic tissues.
